# Ammonia stress-induced heat shock factor 1 enhances white spot syndrome virus infection by targeting the interferon-like system in shrimp

**DOI:** 10.1128/mbio.03136-23

**Published:** 2024-02-15

**Authors:** Xin-Xin Wang, Hui Zhang, Jie Gao, Xian-Wei Wang

**Affiliations:** 1 Shandong Provincial Key Laboratory of Animal Cells and Developmental Biology, School of Life Sciences, Shandong University, Qingdao, Shandong, China; 2 State Key Laboratory of Microbial Technology, Shandong University, Qingdao, Shandong, China; University of California Irvin, Irvine, California, USA

**Keywords:** shrimp, white spot syndrome virus, heat shock factor, interferon, ammonia

## Abstract

Disease emergence is the consequence of host-pathogen-environment interactions. Ammonia is a key stress factor in aquatic environments that usually increases the risk of pathogenic diseases in aquatic animals. However, the molecular regulatory mechanisms underlying the enhancement of viral infection following ammonia stress remain largely unknown. Here, we found that ammonia stress enhances white spot syndrome virus infection in kuruma shrimp (*Marsupenaeus japonicus*) by targeting the antiviral interferon-like system through heat shock factor 1 (Hsf1). Hsf1 is an ammonia-induced transcription factor. It regulates the expression of *Cactus* and *Socs2*, which encode negative regulators of NF-κB signaling and Jak/Stat signaling, respectively. By inhibiting these two pathways, ammonia-induced Hsf1 suppressed the production and function of MjVago-L, an arthropod interferon analog. Therefore, this study revealed that Hsf1 is a central regulator of suppressed antiviral immunity after ammonia stress and provides new insights into the molecular regulation of immunity in stressful environments.

**IMPORTANCE:**

Ammonia is the end product of protein catabolism and is derived from feces and unconsumed foods. It threatens the health and growth of aquatic animals. In this study, we demonstrated that ammonia stress suppresses shrimp antiviral immunity by targeting the shrimp interferon-like system and that heat shock factor 1 (Hsf1) is a central regulator of this process. When shrimp are stressed by ammonia, they activate Hsf1 for stress relief and well-being. Hsf1 upregulates the expression of negative regulators that inhibit the production and function of interferon analogs in shrimp, thereby enhancing white spot syndrome viral infection. Therefore, this study, from a molecular perspective, explains the problem in the aquaculture industry that animals living in stressed environments are more susceptible to pathogens than those living in unstressed conditions. Moreover, this study provides new insights into the side effects of heat shock responses and highlights the complexity of achieving cellular homeostasis under stressful conditions.

## INTRODUCTION

Heat shock factors (HSFs) are a family of transcription factors that control the heat shock response (HSR), which is a highly conserved transcriptional response in all eukaryotes from yeast to humans (1, 2). HSR was originally characterized as a biochemical response that maintains protein homeostasis under heat stress (3). This protection is achieved by upregulating the production of heat shock proteins (HSPs) and molecular chaperones that prevent the aggregation or accumulation of misfolded or damaged proteins (4). Many stressors have proteotoxic consequences; therefore, HSR is not exclusively responsive to thermal stress (5). A wide variety of intrinsic and environmental stress conditions, including cancer, oxidative stress, hypoxia, toxins, and infection, can activate the HSR (6). Therefore, the HSR is a general mechanism for successful survival under stressful conditions.

There is only one HSF in yeast and other invertebrates, while the human genome encodes six paralogs (7, 8). Among the six human (h) HSFs, h-HSF1 has been implicated as the master regulator of HSR (9). h-HSF1 is constitutively expressed in most cells and tissues, and deletion of the *h-hsf1* gene abolishes its ability to upregulate heat-responsive genes and develop thermotolerance after heat shock (10). Activation of h-HSF1 is tightly controlled. Under normal cellular conditions, h-HSF1 is restricted to its inactive form in the cytoplasm via weak interactions with HSP90 and other cochaperones (11). Under stress, the complex is disrupted, releasing h-HSF1. Free monomeric h-HSF1 undergoes trimerization, which is regulated by several post-translational modifications such as phosphorylation and acetylation (12, 13). Active h-HSF1 enters the nucleus, where it binds heat shock elements (HSEs) in the promoters of target genes and initiates transcription (14, 15). h-HSF1-targeted genes include classical heat shock genes (*hsp60*, *hsp70*, *hsp90*, etc.) and also a broad array of genes beyond the scope of molecular chaperones (16). Therefore, h-HSF1 is involved in multiple processes other than HSR, including metabolism (17), immunity (18), and disease (19, 20).

Ammonia is a natural by-product of animal metabolism and a principal stressor in aquatic systems. Although aquatic animals, including fish, crustaceans, and shellfish, show varied levels of tolerance to ammonia, it is clear that both acute stress and chronic ammonia stress increase the risk of diseases caused by pathogens in multiple aquatic species (21, 22). Ammonia is toxic to aquatic animals as it influences osmoregulation, suppresses oxygen uptake, damages tissue integrity, and causes many other physiological, biochemical, and pathological manifestations (23, 24). However, the molecular regulatory mechanism by which ammonia stress enhances viral infection remains largely unknown.

In this study, we found that ammonia stress enhanced white spot syndrome virus (WSSV) infection in the kuruma shrimp (*Marsupenaeus japonicus*), an important aquaculture species that continuously accumulates ammonia in water. By screening for ammonia-induced factors using RNA sequencing (RNA-seq) and investigating their regulatory responses, we aimed to uncover the molecular mechanism by which ammonia stress enhances WSSV infection. The identification and characterization of Hsf1 in this process provide a molecular perspective on the mechanism by which environmental stress affects host immunity.

## RESULTS

### Ammonia stress enhances WSSV infection

An appropriate concentration of ammonia was first determined for the stress treatment. Based on the available information (25), the total ammonia concentration in the culture water was adjusted to be 0.5, 1, 2, 5, 10, or 20 mg/L. When shrimp were treated with acute ammonia stress for 12 h before WSSV infection, the transcription level of *vp28*, which encodes the most abundant envelope protein in WSSV and therefore can be used to monitor the level of WSSV infection, increased when the culture water contained 1, 2, 5, or 10 mg/L of total ammonia. However, 20 mg/L ammonia suppressed *vp28* expression (Fig. S1A). The suppression of WSSV infection may have been caused by physiological disorders (e.g., increase in oxidative stress and damage to tissue integrity) in shrimps at a relatively high concentration of ammonia in the culture water. To clarify whether ammonia stress damages the host physiology, we measured the total antioxidant capacity, catalase activity, superoxide dismutase activity, and glutathione peroxidase activity in the hepatopancreas, which are important indicators of host physiology (26, 27). As shown in Fig. S1B, all activities were elevated by ammonia stress at 10 and 20 mg/L but not at 5 mg/L. Moreover, shrimp tissue integrity did not change in the 2 and 5 mg/L groups but showed changes reflected mainly in the spread of hepatopancreatic tubule lumens in the 20 mg/L group (Fig. S1C). These findings suggest that low concentrations of ammonia (1, 2, and 5 mg/L) did not significantly damage host physiology and that the enhanced WSSV infection may be caused by specific molecular mechanisms after ammonia stress. Therefore, a concentration of 5 mg/L was selected for subsequent experiments.

**Fig 1:**
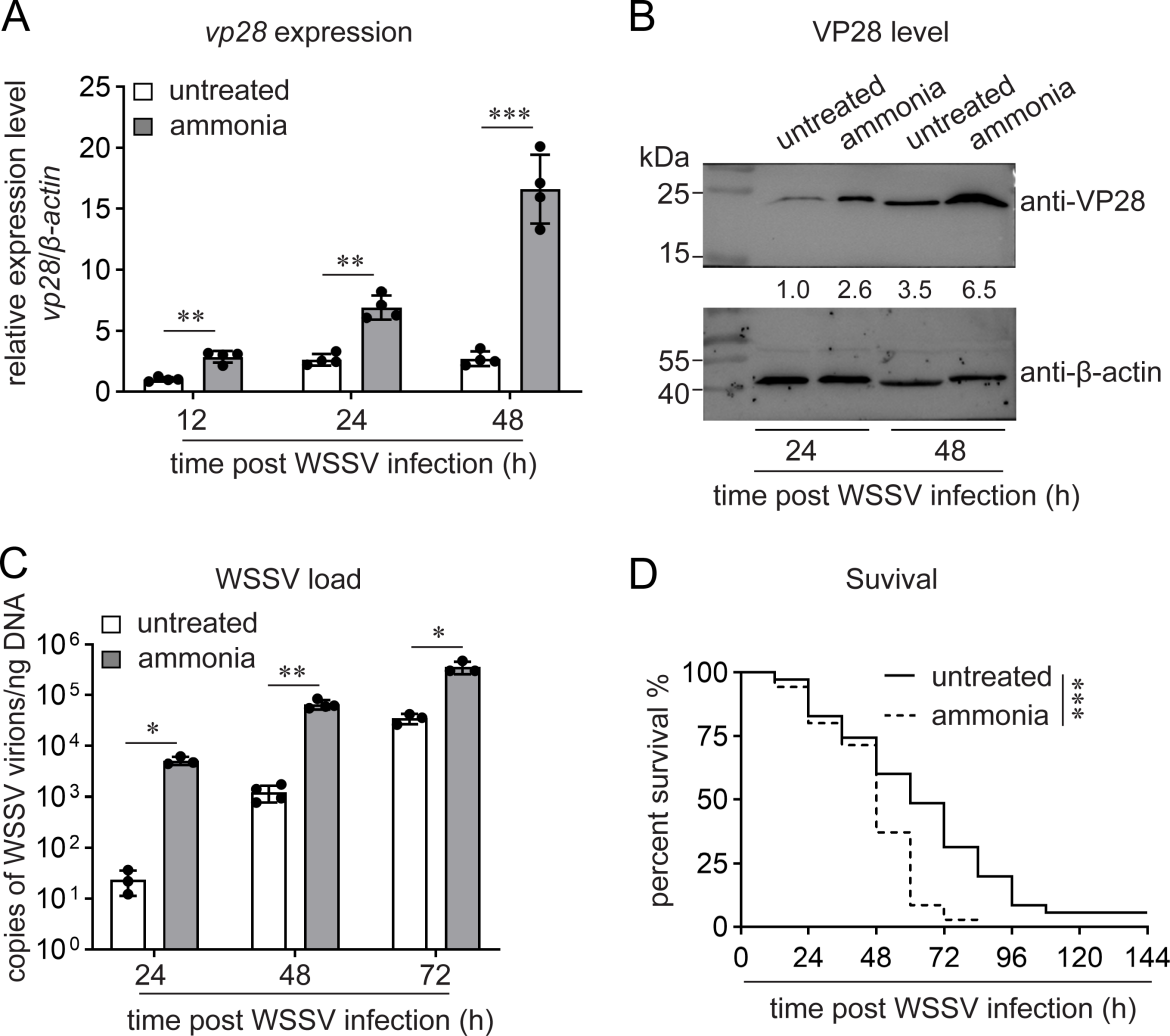
Enhancement of WSSV infection by ammonia stress. (**A–C**) Enhancement of WSSV infection by ammonia stress. Shrimp were transferred into seawater containing 5 mg/L of total ammonia nitrogen (TAN), while the control animals were cultured in normal seawater. WSSV infection (5 × 10^5^ virions) was performed 12 h later. *vp28* expression (**A**), VP28 levels (**B**), and viral load (**C**) in shrimp gills were detected at the indicated times. (**D**) Acceleration of WSSV-induced shrimp death by ammonia stress. The shrimp survival rate was recorded after WSSV infection (10^6^ virions) every 12 h for 6 d. *n* = 35, log-rank (Mantel–Cox) test. Bar chart data are shown as the mean ± standard deviation (SD) from three or four replicates. ∗∗∗*P* < 0.001, ∗∗0.001 < *P* < 0.01, and ∗*P* < 0.05, as determined using Student’s *t*-test. Western blot images are representative of three independent replicates. At least five shrimp were used to prepare each sample.

We confirmed the enhancement of WSSV infection caused by ammonia stress. As shown in Fig. 1A and B, 5 mg/L of ammonia stress led to increased *vp28* transcription and VP28 production at 24 and 48 h after WSSV infection. In addition, the viral load was significantly higher in the ammonia-stressed shrimp than in the untreated shrimp (Fig. 1C). Moreover, the survival rate after WSSV infection was consistently lower in the stressed group than that in the control group (Fig. 1D). These results demonstrated that ammonia stress enhances WSSV infection.

### Hsf1 is a key factor for ammonia-enhanced WSSV infection

**Fig 2:**
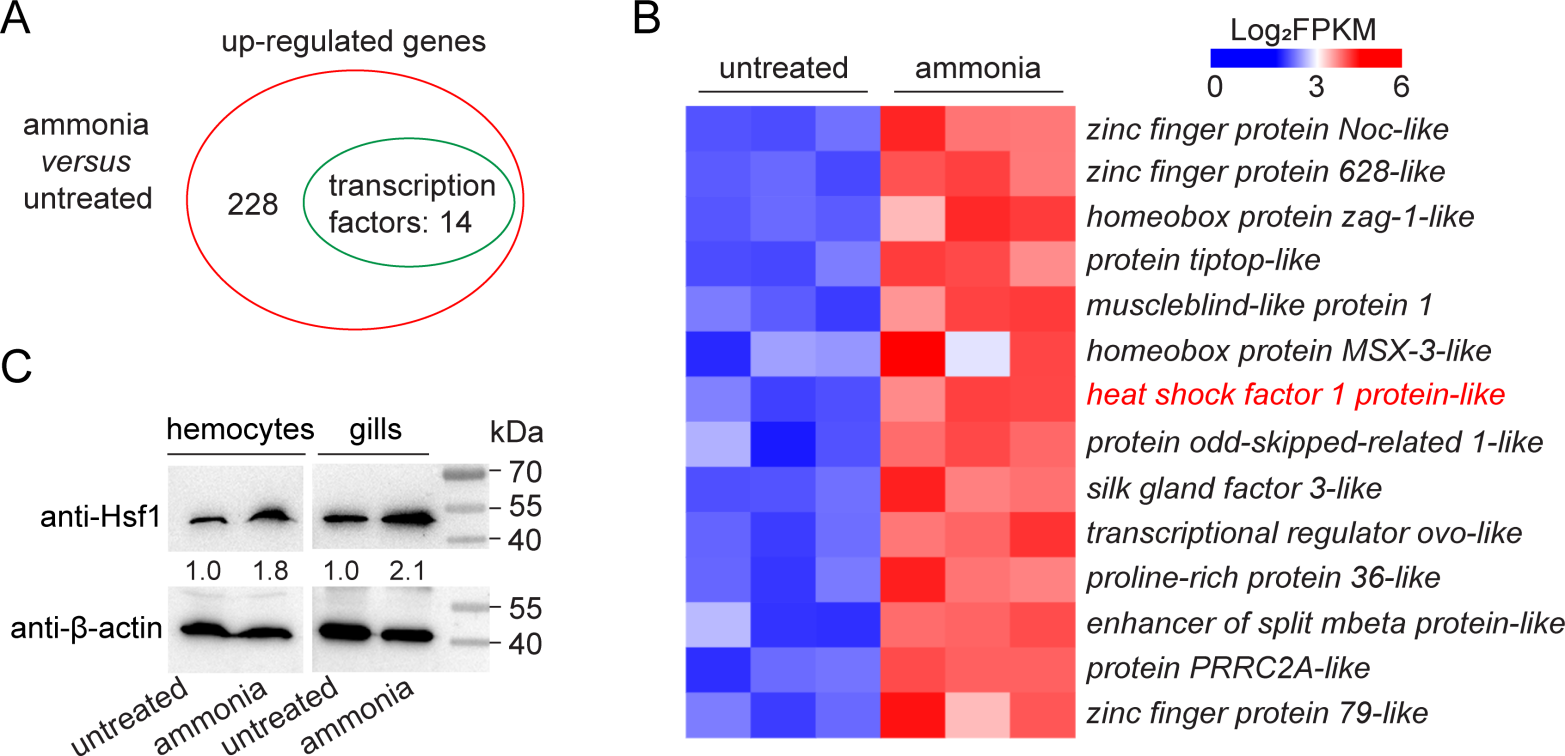
Identification of Hsf1 as a key factor induced by ammonia stress. (**A**) Identification of ammonia-induced genes by RNA-seq. RNA-seq was performed 12 h after ammonia stress treatment. *n* = 30. (**B**) Heat map showing the expression levels of differentially expressed genes which encode potential transcription factors. The fragments per kilobase per transcript per million mapped reads (FPKM) values from three biological replicates are shown after taking the logarithm. (**C**) Expression profiles of Hsf1 after ammonia stress treatment. Shrimp were cultured in normal seawater or seawater containing 5 mg/L TAN. Hsf1 levels in the indicated tissues were determined using western blotting. Blot data are representative of three independent replicates. At least five shrimp were used to prepare each sample.

To reveal the molecular mechanism by which ammonia stress enhances WSSV infection, RNA-seq was performed to screen for genes that respond to ammonia stress, particularly those that encode potential transcriptional regulators. As shown in Fig. 2A and B, 14 of the 228 ammonia-induced genes were predicted to encode transcription factors. In addition, no transcription factors were predicted among the genes whose expression was suppressed by ammonia stress. Special attention has been paid to shrimp Hsf1 because of the wide involvement of its human counterpart in the stress response (6). To validate the high-throughput sequencing results, the expression profile of shrimp *hsf1* was determined by reverse transcription polymerase chain reaction (RT-PCR) and western blotting. *hsf1* mRNA was detected in all the tested tissues (Fig. S2A). Ammonia stress induced *hsf1* transcription and Hsf1 production significantly in the gills and hemocytes (Fig. 2C; Fig. S2B). To determine whether Hsf1 is related to WSSV infection, its expression level after WSSV infection was also determined. The results showed that *hsf1* expression levels increased from 6 to 24 h after WSSV infection (Fig. S2C). Induction by both ammonia and WSSV suggested that Hsf1 may be involved in the molecular regulation of ammonia-enhanced WSSV infection.

**Fig 3:**
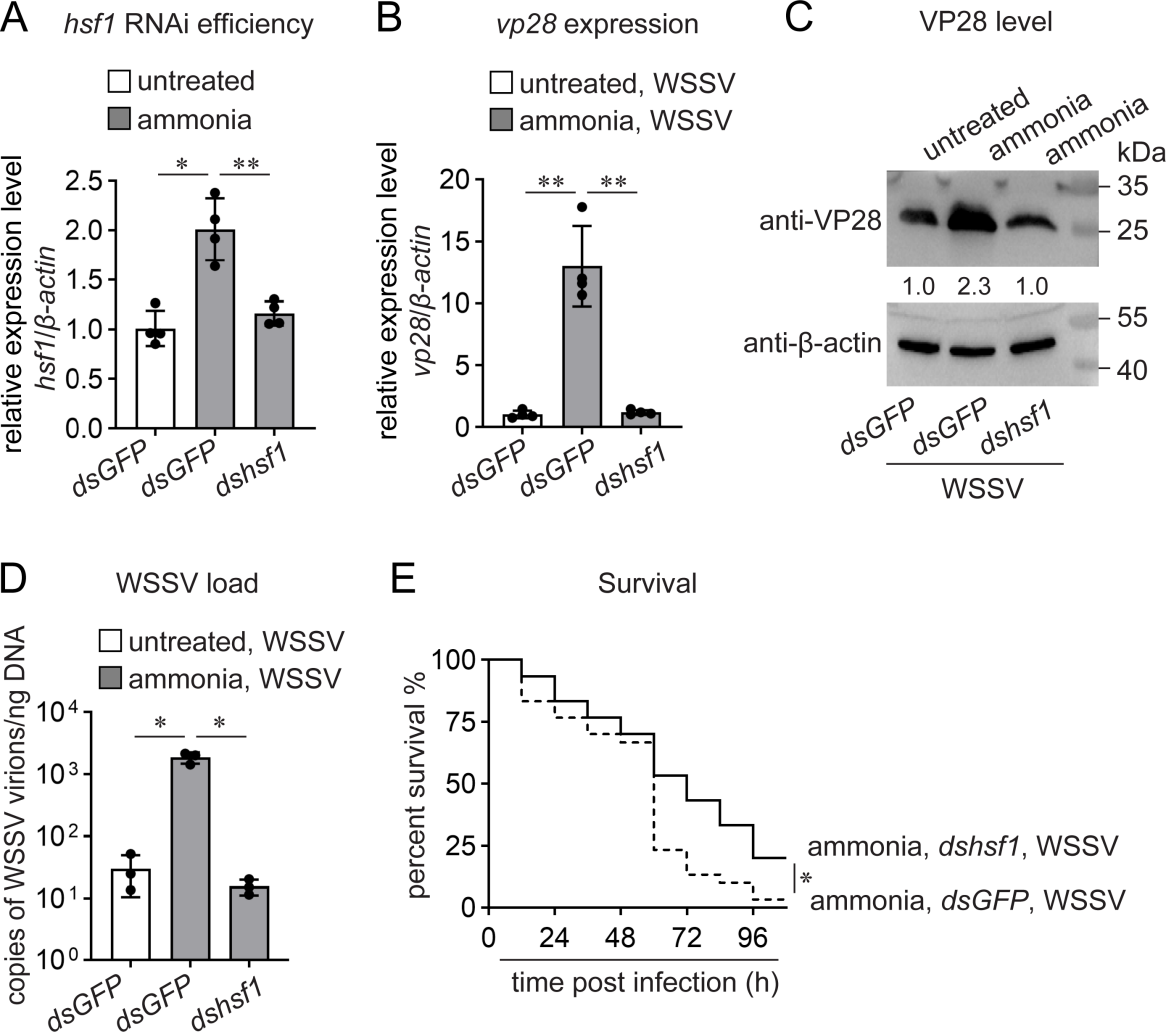
Role of Hsf1 in ammonia-enhanced WSSV infection. (**A**) Efficiency of *hsf1* RNA interference (RNAi). Shrimp were maintained in normal seawater or transferred into seawater containing 5 mg/L TAN 36 h after double-stranded (ds) RNA injection. *hsf1* expression was detected 12 h later using quantitative PCR (qPCR). (**B–D**) Suppression of ammonia-enhanced WSSV infection by *hsf1* knockdown. WSSV infection (5 × 10^5^ virions) was performed 48 h after dsRNA injection. *vp28* expression (**B**), VP28 levels (**C**), and viral load (**D**) in shrimp gills were determined 24 h later. (**E**) Decrease in ammonia/WSSV-induced shrimp death by *hsf1* knockdown. The shrimp survival rate was recorded every 12 h after WSSV infection (10^6^ virions). *n* = 30, log-rank (Mantel–Cox) test. Bar chart data are shown as the mean ± SD from three or four replicates. ∗∗0.001 < *P* < 0.01 and ∗*P* < 0.05, as determined using Student’s *t*-test. Blot data are representative of three independent replicates. At least five shrimp were used to prepare each sample.

To test this hypothesis, we evaluated WSSV infection levels after silencing *hsf1* expression (Fig. 3A). As shown in Fig. 3B and C, *hsf1* knockdown reduced ammonia-mediated enhancement of both *vp28* transcription and VP28 production. In addition, the increase in viral load induced by ammonia was suppressed by *hsf1* knockdown (Fig. 3D). Moreover, the survival rate after ammonia and WSSV treatment was higher in the *hsf1*-knockdown group than that in the control group (Fig. 3E). Together, these data showed that Hsf1 is indeed a key factor in enhancing WSSV infection after ammonia stress.

### Ammonia-induced Hsf1 targets the interferon-like system to enhance WSSV infection

**Fig 4:**
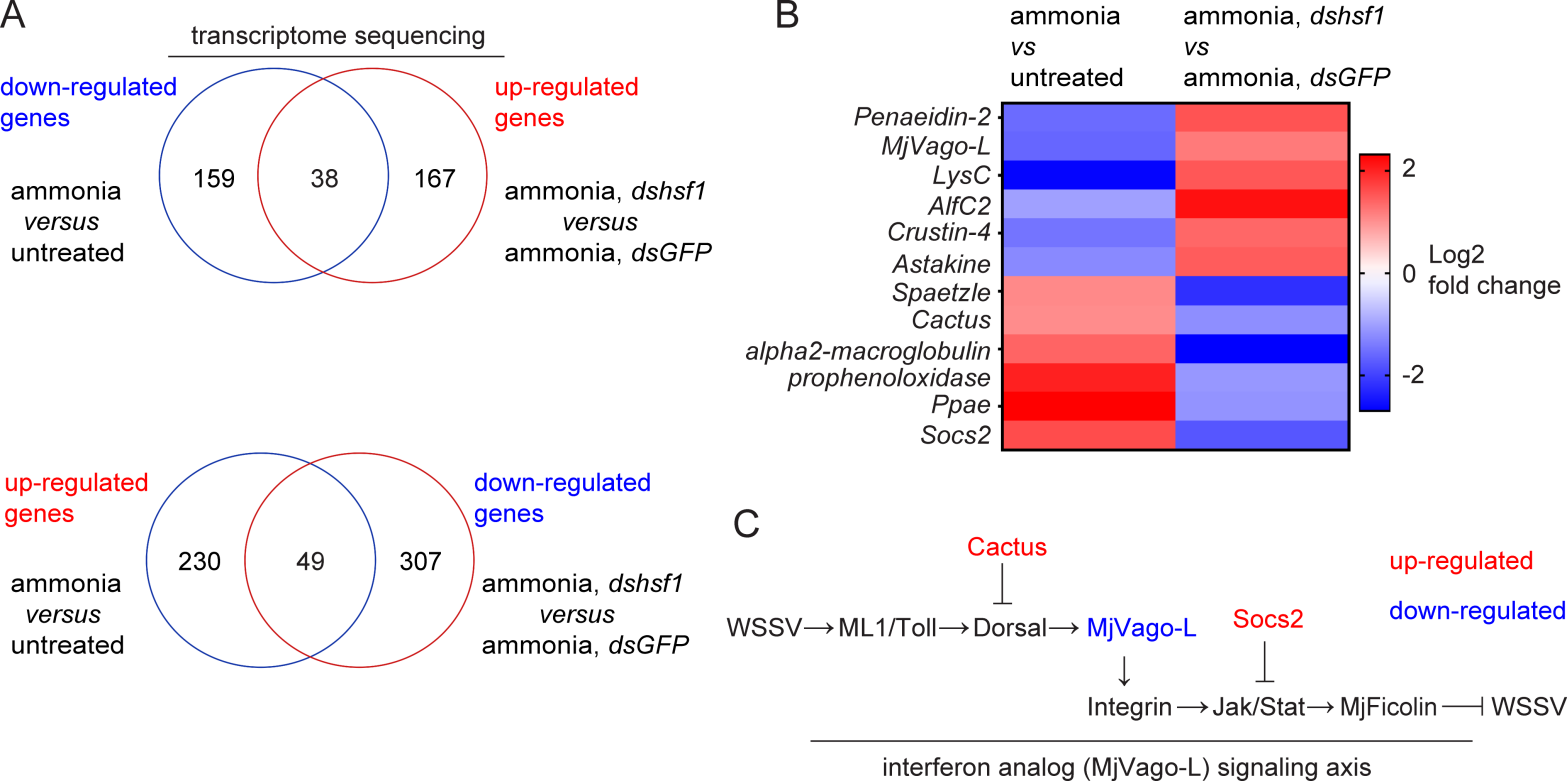
Possible regulation of the interferon-like system by ammonia stress. (**A**) Venn diagram of the overlap of differentially expressed genes. RNA-seq was performed at 12 h after ammonia stress treatment or 48 h after dsRNA injection (dsRNA injection was performed 36 h before ammonia stress treatment). *n* = 30. (**B**) Heat map showing the expression levels of differentially expressed transcription factors. The fold change of each gene is shown after taking the logarithm. (**C**) Illustration of the shrimp interferon-like system. The interferon analog, MjVago-L, is synthesized through NF-κB/Dorsal signaling and plays an antiviral role by activating Jak/Stat signaling and inducing the antiviral effector MjFicolin. Cactus and Socs2 are negative regulators of NF-κB/Dorsal signaling and Jak/Stat signaling, respectively.

To determine how ammonia-induced Hsf1 enhances WSSV infection, we performed RNA-seq to screen for the target genes regulated by ammonia-induced Hsf1. We aimed to identify candidates whose expression was induced or inhibited by ammonia stress and whose induction or inhibition was reversed by *hsf1* knockdown. Taking the intersection of the differentially expressed genes (DEGs) from the two datasets generated 38 candidates that were suppressed by ammonia-induced Hsf1 and 49 candidates that were induced by ammonia-induced Hsf1 (Fig. 4A). These DEGs comprised a set of immunity-related genes, including antimicrobial peptides, cytokines, serine proteases, and inhibitors and regulators of immune signaling pathways (Fig. 4B). Interestingly, the expression of shrimp interferon analog *MjVago-L* (Vago-like), which was characterized in our previous studies, was suppressed by ammonia-induced Hsf1, while the expression of *Cactus* and *Socs2*, which encode two negative regulators of nuclear factor kappa B (NF-κB)/Dorsal signaling and Janus kinase (Jak)/signal transducer and activator of transcription (Stat) signaling (28, 29), was induced by ammonia-induced Hsf1. Previously, we established that the interferon analog MjVago-L is synthesized through NF-κB/Dorsal signaling and plays an antiviral role by activating Jak/Stat signaling (30, 31), suggesting that Hsf1 may target the interferon-like system by inducing negative regulators, thereby suppressing the production and function of the interferon analog (Fig. 4C).

**Fig 5:**
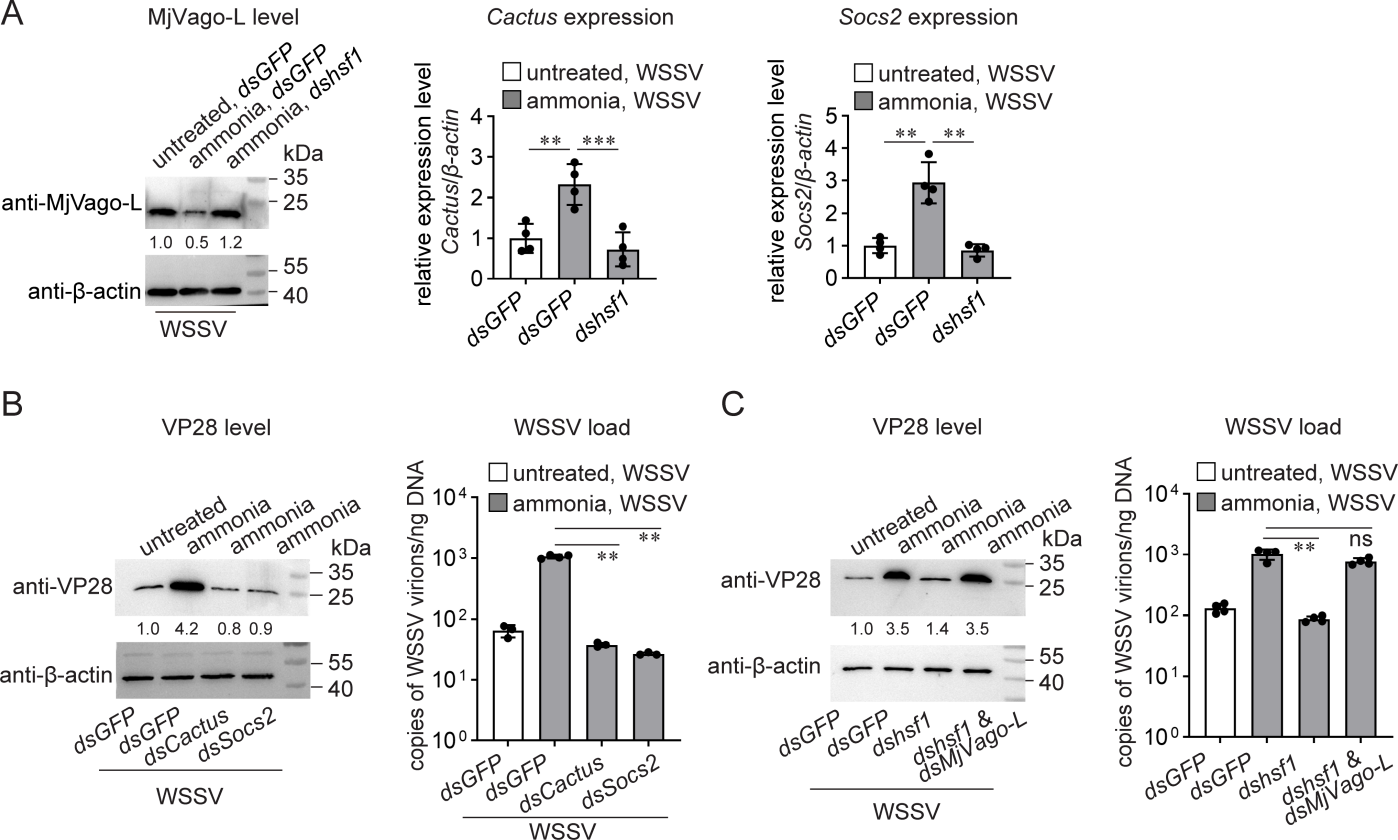
Contribution of the interferon-like system components to ammonia-enhanced WSSV infection. (**A**) Regulation of the production of MjVago-L and the expression of *Cactus* and *Socs2* by ammonia-induced Hsf1. Shrimp were maintained in normal seawater or transferred to seawater containing 5 mg/L TAN 36 h after dsRNA injection. WSSV infection was performed 12 h later. Gene expression was determined using qRT-PCR, and protein levels were analyzed using western blotting 24 h later. (**B**) Suppression of ammonia-enhanced WSSV infection by *Cactus* or *Socs2* knockdown. dsRNA was injected 36 h before ammonia stress treatment. WSSV infection was performed 48 h after dsRNA injection. The VP28 levels and viral load in the shrimp gills were detected 24 h later. (**C**) Restoring ammonia-enhanced WSSV infection by *MjVago-L* knockdown. dsRNA was injected at 36 h before ammonia stress treatment. WSSV infection was performed 48 h after dsRNA injection. The VP28 levels and viral load in the shrimp gills were detected 24 h later. Bar chart data are shown as the mean ± SD from three or four replicates. ∗∗∗*P* < 0.001, ∗∗0.001 < *P* < 0.01, and ∗*P* < 0.05, as determined using Student’s *t*-test. Blot data are representative of three independent replicates. At least five shrimp were used to prepare each sample.

Next, we confirmed the RNA-seq results by qRT-PCR and western blotting. As shown in Fig. 5A, MjVago-L production was inhibited by ammonia stress, and this inhibition was abolished by *hsf1* knockdown. In contrast, the induction of both *Cactus* and *Socs2* transcriptions by ammonia stress was blocked when *hsf1* expression was silenced.

To determine whether the regulation of *Cactus*, *Socs2*, and *MjVago-L* by ammonia-induced Hsf1 contributed to enhanced WSSV infection, the roles of these factors were analyzed. The results showed that both Cactus and Socs2 are important for ammonia-induced Hsf1-enhanced WSSV infection. Knockdown of either *Cactus* or *Socs2* transcription led to lower VP28 production and viral load than those in the control group (Fig. 5B). Moreover, inhibition of MjVago-L was essential for ammonia-induced Hsf1-enhanced WSSV infection. Enhanced WSSV infection, which was suppressed by *hsf1* knockdown, was restored by the simultaneous silencing of *hsf1* and *MjVago-L* (Fig. 5C). These data suggested that ammonia-induced Hsf1 enhanced WSSV infection by inhibiting the shrimp interferon-like system.

### Ammonia-induced Hsf1 suppresses the sequential Dorsal/MjVago-L/Stat axis by inducing Cactus and Socs2

**Fig 6:**
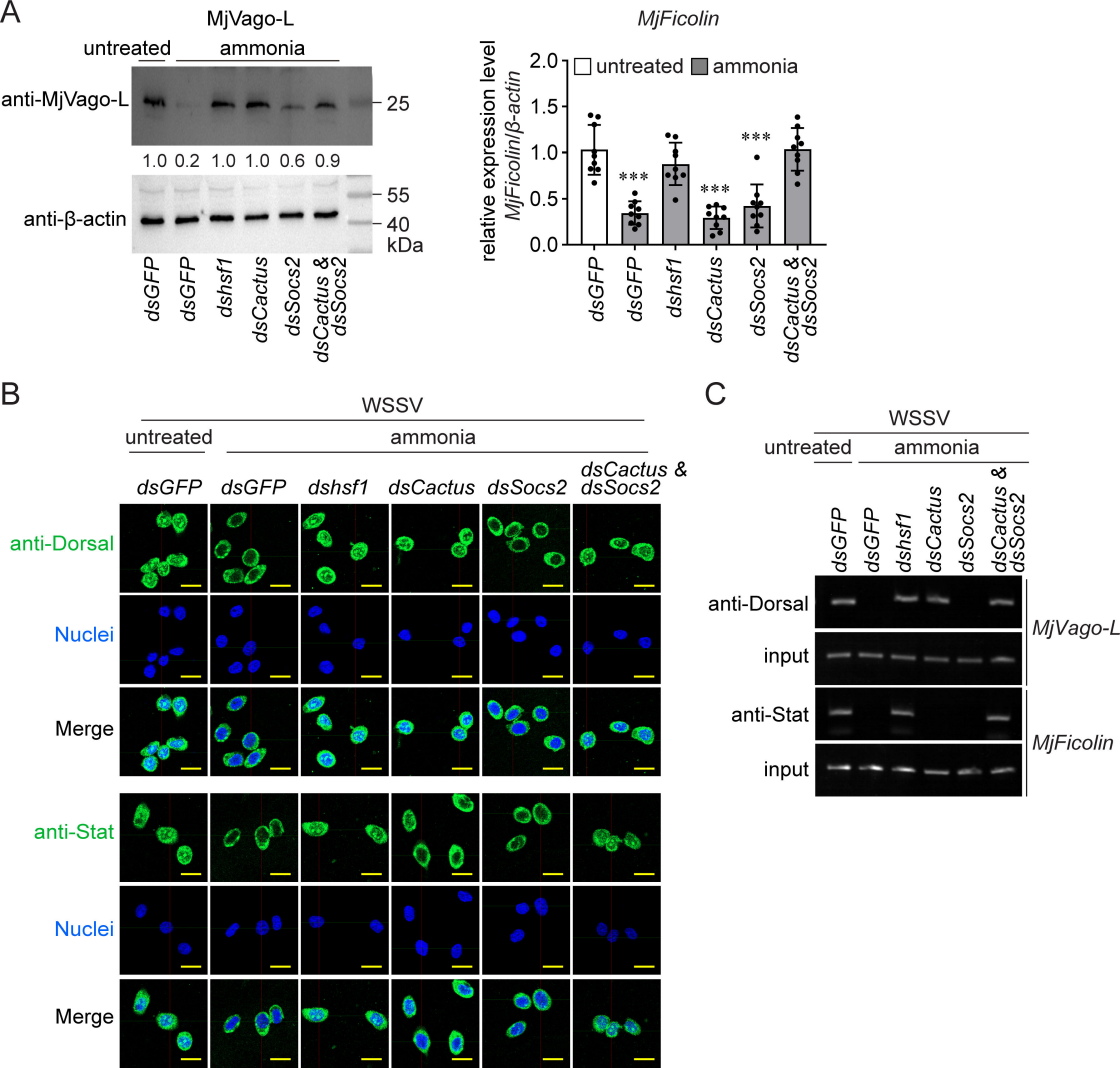
Inhibition of the sequential Dorsal/MjVago-L/Stat axis by ammonia stress. (**A**) Regulation of MjVago-L production and *MjFicolin* expression by ammonia-induced Hsf1-induced Cactus and Socs2. Shrimp were injected with the indicated dsRNAs and stressed by ammonia 36 h later. Gene expression was detected another 12 h later, while protein production was detected another 24 h later. Bar chart data show the mean ± SD from nine individuals. ∗∗∗*P* < 0.05, as determined using Student’s *t*-test. The blotting images are representatives of three replicates. (**B**) Regulation of the nuclear levels of Dorsal and Stat by ammonia-induced Hsf1-induced Cactus and Socs2. Shrimp were injected with the indicated dsRNAs and stressed by ammonia 36 h later. WSSV infection was performed another 12 h later. An immunocytochemical assay was performed to detect the subcellular distribution of Dorsal and Stat 24 h after WSSV infection. Scale bar = 10 μm. (**C**) Regulation of Dorsal/*MjVago-L* and Stat/*MjFicolin* transcription by ammonia-induced Hsf1-induced Cactus and Socs2. Images are representative of three or four replicates. At least five shrimp were used to prepare each sample.

The detailed regulatory mechanism by which ammonia-induced Hsf1 inhibited the interferon-like system was next elucidated. We measured the expression levels of MjVago-L and MjFicolin, the final antiviral effector downstream of the MjVago-L/Jak/Stat axis, to evaluate the activity of Dorsal and Stat transcription factors specifically targeted by Cactus and Socs2, respectively. As shown in Fig. 6A, knockdown of *hsf1* inhibited the ammonia-mediated suppression of both MjVago-L production and *MjFicolin* expression. Knockdown of *Cactus*, but not *Socs2*, restored MjVago-L production, whereas knockdown of either inhibitor did not restore *MjFicolin* expression. Simultaneous knockdown of *Cactus* and *Socs2* restored *MjFicolin* expression. These data indicate a probable sequential regulation of the Dorsal/MjVago-L/Stat/MjFicolin axis by ammonia-induced Hsf1 by targeting two nodes: Dorsal and Stat.

Activated Dorsal and Stat are enriched in the nucleus to promote the transcription of *MjVago-L* and *MjFicolin*, respectively (30, 31). Therefore, we determined the subcellular locations of Dorsal and Stat to further elucidate the regulatory mechanisms. Immunocytochemical analysis revealed that ammonia stress decreased the amount of Dorsal and Stat in the nucleus after WSSV infection. This suppression was reversed by *hsf1* knockdown. The decrease of Dorsal in the nucleus was blocked by *Cactus* knockdown but not *Socs2* knockdown, whereas the decrease in Stat levels was not reversed by either treatment. Moreover, the simultaneous knockdown of *Cactus* and *Socs2* reversed the decrease in both Dorsal and Stat in the nucleus (Fig. 6B; Fig. S3A). Immunocytochemical results were confirmed by separating and analyzing the nuclear proteins after the indicated treatments (Fig. S4A). This suggests that ammonia-induced Hsf1 targets Dorsal and Stat by inducing the production of Cactus and Socs2, respectively, and that Stat functions downstream of Dorsal in sequential regulation.

Because Dorsal and Stat can directly regulate *MjVago-L* and *MjFicolin* transcription, respectively, a chromatin immunoprecipitation (ChIP) assay was performed to confirm the regulatory mechanism. As shown in Fig. 6C, ammonia stress led to a decrease in the amount of *MjVago-L* and *MjFicolin* promoter fragments in the immunoprecipitates obtained using anti-Dorsal and anti-Stat antibodies, respectively. As expected, *hsf1* knockdown restored the levels of both *MjVago-L* and *MjFicolin* fragments. Knockdown of *Cactus* alone suppressed the decrease in *MjVago-L* fragments, whereas the simultaneous knockdown of *Cactus* and *Socs2* suppressed the decrease in *MjFicolin* fragments. These data also support the sequential Dorsal/MjVago-L/Stat/MjFicolin axis.

Next, recombinant MjVago-L, which was found to be effective *in vivo* for antiviral immunity (31), was used to confirm whether MjVago-L is indeed the key intermediate in ammonia-induced Hsf1 downstream sequential regulation. As shown in Fig. S5A, the ammonia-induced downregulation of *MjFicolin* (which was not identified in the RNA-seq data because of the cut-off fold change of ≥2) was abolished by exogenous MjVago-L application. We also examined the effects of MjVago-L on ammonia-enhanced WSSV infection. The results showed that VP28 production was significantly lower after exogenous MjVago-L application than in the control group (Fig. S5B). Moreover, exogenous MjVago-L treatment increased shrimp survival following the ammonia/WSSV treatment (Fig. S5C). Therefore, the above data collectively show that inhibition of the sequential Dorsal/MjVago-L/Stat axis contributes to ammonia-enhanced WSSV infection.

### p38 is essential for ammonia-induced Hsf1 activation

**Fig 7:**
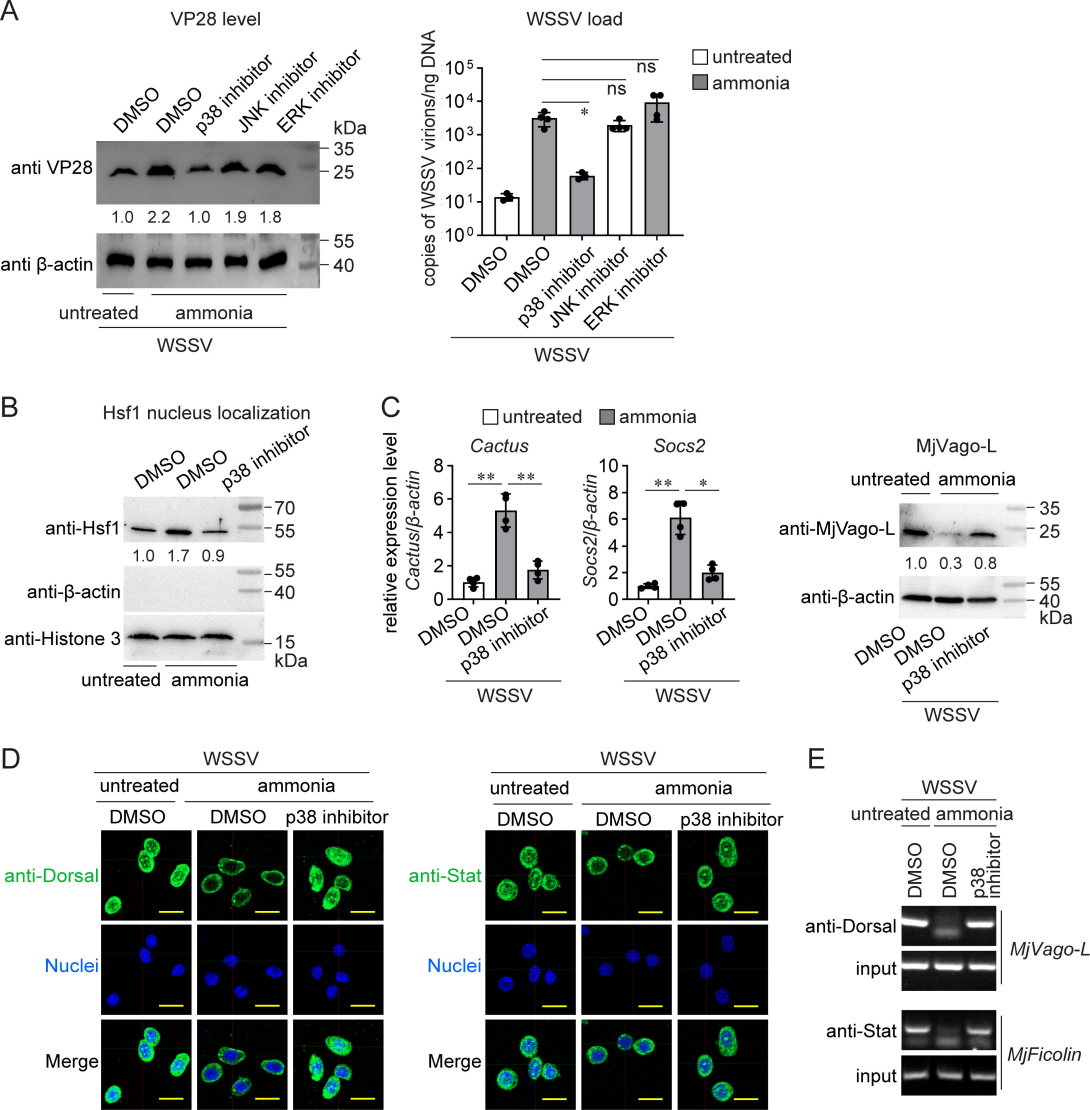
P38 is required for ammonia-enhanced WSSV infection. (**A**) Suppression of ammonia-enhanced WSSV infection by p38 inhibition. Shrimp were maintained in normal seawater or subjected to ammonia stress. The inhibitors were then administered 30 min later. WSSV infection was performed after 12 h. VP28 levels and viral loads were measured in the gills. (**B**) Suppression of ammonia-induced Hsf1 activation by p38 inhibition. Shrimp were maintained in normal seawater or subjected to ammonia stress. The inhibitor was then administered 30 min later. The nuclear levels of Hsf1 in the gills were determined 2 h later. (**C**) Suppression of ammonia-mediated changes in gene expression caused by p38 inhibition. Shrimp were maintained in normal seawater or subjected to ammonia stress. The inhibitors were then administered 30 min later. WSSV infection was performed after 12 h. Gene expression and protein production levels were determined 24 h after infection. (**D**) Suppression of ammonia-reduced nuclear levels of Dorsal and Stat by p38 inhibition. Immunocytochemical analyses were performed 24 h after WSSV infection using the indicated antibodies. Scale bar = 10 μm. (**E**) Suppression of ammonia-reduced Dorsal/*MjVago-L* transcription and Stat/*MjFicolin* transcription by p38 inhibition. ChIP assays were performed 24 h after WSSV infection. All bar charts show the mean ± SD from three replicates. ∗∗∗*P* < 0.001, ∗∗0.001 < *P* < .01, and ∗*P* < 0.05, as determined using Student’s *t*-test. Images are representative of three or four replicates. At least five shrimp were used to prepare the samples.

We showed that ammonia stress induced *hsf1* expression to suppress shrimp immunity. However, how Hsf1 activity is regulated by ammonia is unclear. Hsf1 translocates to the nucleus after activation (9), and mitogen-activated protein kinases (MAPKs) are activated by diverse extracellular stimuli, including heat shock (32). Therefore, we determined whether any MAPK in shrimp was responsible for ammonia-suppressed antiviral immunity. Specific inhibitors of p38, c-Jun N-terminal kinase (JNK), and extracellular regulated kinase (ERK), the three classical MAPKs, were used after ammonia treatment. As shown in Fig. 7A, p38 inhibition abolished ammonia-enhanced WSSV infection, as revealed by VP28 production and viral load, whereas JNK and ERK inhibition did not, suggesting that p38 may be a key MAPK involved in ammonia-suppressed immunity. Therefore, we evaluated p38 activation by assessing p38 phosphorylation following ammonia stress. The result showed that ammonia stress induced p38 phosphorylation (Fig. S6). Next, we investigated whether p38 was responsible for Hsf1 activation. The results revealed that ammonia increased the amount of Hsf1 in the nucleus, and p38 inhibition decreased the amount of Hsf1 (Fig. 7B).

Next, the regulation of the Hsf1-downstream Dorsal/MjVago-L/Stat axis by p38 was demonstrated. As shown in Fig. 7C, the induction of *Cactus* and *Socs2* after ammonia treatment was blocked by p38 inhibition. Moreover, the decrease in the amount of activated Dorsal and Stat in the nucleus following ammonia treatment was attenuated by p38 inhibition (Fig. 7D; Fig. S3B and S4B). In addition, application of a p38 inhibitor enhanced the binding of Dorsal and Stat to the *MjVago-L* and *MjFicolin* promoters, respectively (Fig. 7E), ultimately restoring MjVago-L production (Fig. 7C). Therefore, these data demonstrate that p38 MAPK is essential for ammonia-induced Hsf1 activation and that ammonia-induced Hsf1 suppressed antiviral immunity.

**Fig 8:**
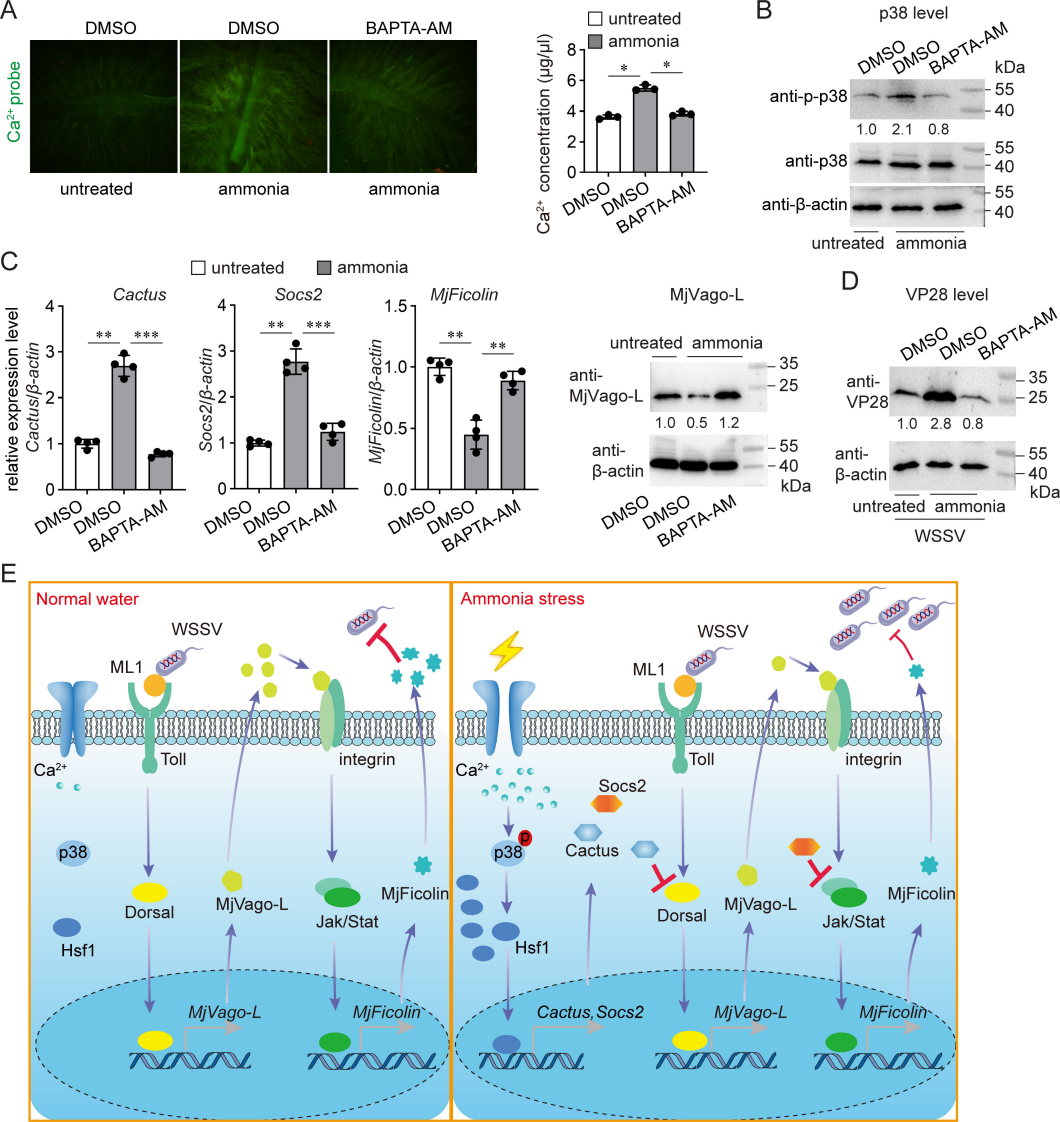
Involvement of calcium influx in ammonia-enhanced WSSV infection. (**A**) Induction of calcium influx in gills by ammonia stress treatment. Shrimp were maintained in normal seawater or subjected to ammonia stress treatment. The calcium chelator BAPTA-AM was administered 30 min later. After another 30 min, the tissues were stained with the calcium fluorescence probe Fluo-3AM and observed under a confocal microscope (left panel). The intracellular calcium concentration was determined using a calcium colorimetric assay kit (right panel). (**B**) Suppression of ammonia-induced p38 activation by calcium chelation. Shrimp were maintained in normal seawater or subjected to ammonia stress treatment. BAPTA-AM or the control treatment was administered 30 min later. The p38 levels in the gills were determined 12 h later. (**C**) Suppression of ammonia-induced gene expression variation by calcium chelation. Shrimp were maintained in normal seawater or subjected to ammonia stress treatment. BAPTA-AM or the control treatment was administered 30 min later. Gene expression levels were determined another 12 h later, while protein production levels were determined another 24 h later. (**D**) Suppression of ammonia-enhanced WSSV infection by calcium chelation. Shrimp were maintained in normal seawater or subjected to ammonia stress treatment. BAPTA-AM or the control treatment was administered 30 min later. WSSV infection was performed another 12 h later. The VP28 levels in the gills were determined 24 h after infection. (**E**) Working model of this study. Ammonia stress induces the production of Hsf1, leading to calcium influx, which activates p38 and Hsf1. Activated Hsf1 induces the production of Cactus and Socs2, which target the shrimp interferon-like system at two nodes, namely, Dorsal and Stat. By suppressing shrimp antiviral immunity through inhibiting the production and function of MjVago-L, ammonia stress enhances WSSV infection. All bar charts show the mean ± SD from three replicates. ∗∗∗*P* < 0.001, ∗∗0.001 < *P* < .01, and ∗*P* < 0.05, as determined using Student’s *t*-test. Images are representative of three replicates. At least five shrimp were used to prepare the samples.

Next, we determined the mechanism by which ammonia stress leads to p38 activation. Previous studies have shown that ammonia treatment increases intracellular calcium concentration and that intracellular calcium mobilization is an effective inducer of the p38 signaling pathway (33, 34). Therefore, we investigated whether calcium signaling is involved in ammonia-enhanced WSSV infection. Ammonia treatment led to elevated calcium concentrations in shrimp gills, as revealed using a fluorescent probe and colorimetric kit (Fig. 8A). When BAPTA-AM, a cell-permeable chelator, was used to control intracellular calcium mobilization and ammonia-induced p38 activation, the altered expression of *Cactus*, *Socs2*, and *MjFicolin* and the altered production of MjVago-L disappeared (Fig. 8B and C). Moreover, the ammonia-induced increase in VP28 production was abolished (Fig. 8D). Taken together, these results demonstrate that calcium/p38 signaling is induced by ammonia stress and is essential for Hsf1-mediated WSSV infection.

## DISCUSSION

This study provides a molecular explanation for how ammonia stress suppresses host immunity and increases sensitivity to pathogenic infections in aquaculture. Ammonia is one of the major limiting factors in aquaculture. Aquatic animals reared in water with high ammonia levels are more sensitive to pathogens than those reared in water with normal ammonia levels (21, 22). Previous studies have demonstrated that ammonia stress can suppress immune parameters in shrimp, including total hemocyte count, hemocyte phagocytic ability, hemolymph phenoloxidase activity, and plasma antimicrobial activity (24). This mechanism contributes to decreased pathogen resistance in shrimp after exposure to ammonia. However, whether a specific molecular regulatory mechanism exists in addition to the suppression of these immune parameters remains largely unknown. In the present study, we showed that ammonia-activated Hsf1 is essential for increasing the sensitivity of shrimp to WSSV. *Cactus* and *Socs2* are two direct readouts of ammonia-activated Hsf1 signaling. By inducing two negative regulators of Dorsal and Jak/Stat signaling, ammonia-induced Hsf1 specifically targets the production and function of MjVago-L (28, 29). The shrimp interferon-like system is important for antiviral immunity (31); therefore, the inhibition of this system leads to enhanced WSSV infection after ammonia stress. This molecular mechanism, together with previously reported biochemical and physiological mechanisms, revealed how ammonia stress attenuates the shrimp immune response.

By demonstrating that ammonia-activated Hsf1 suppresses antiviral immunity in shrimp, this study provides new insights into the side effects of HSR. HSRs are crucial for stress relief because they are highly conserved among organisms and widely responsive to multiple stresses (2). However, as h-HSF1, the master transcription factor of HSR, can regulate the transcription of many genes beyond stress-related genes, HSR is usually accompanied by certain side effects (4, 16). For example, a recent study showed that mouse Hsf1 controls the expression of heterogeneous nuclear ribonucleoprotein A2/B1, a key regulator of mouse beige fat activation and energy homeostasis. This offers a promising strategy for treating obesity through local hyperthermia therapy (35). In this study, we revealed that when shrimp are stressed by ammonia, the host enhances the HSR to prevent proteotoxicity and global cellular stress and maintain well-being in a stressed environment. Simultaneously, ammonia-activated Hsf1 regulates the expression of immunity-related genes, ultimately leading to biased immunity in shrimp. Therefore, our findings, together with those of other studies, highlight the complexity of achieving cellular homeostasis under stressful conditions.

Multiple studies have shown that Hsf1 is involved in host-virus interactions; however, the current mechanism by which shrimp Hsf1 participates in viral infection is different from that described in humans. Human h-HSF1 is involved in various viral infections. First, h-HSF1 directly regulates viral gene expression. By directly interacting with HSE present in the promoter of the long terminal repeat of human immunodeficiency virus-1 (HIV-1), h-HSF1 enhances viral gene expression and replication (36). A similar situation has been observed for the bovine leukemia virus (37). Secondly, h-HSF1 promotes viral infection by regulating the expression of host genes. For example, after orthopoxvirus infection, the upregulated h-HSF1 is phosphorylated and translocated to the nucleus. Activated h-HSF1 initiates the transcription of HSPs packaged within virions and is used to extend the viral genome (38). In addition, h-HSF1, together with the viral Nef protein, induces the expression of HSP40, which further facilitates viral gene expression (36). Moreover, h-HSF1 mediates HIV-1 latency reversal by regulating stress pathways and promoting viral RNA elongation (39, 40). In contrast, the present study showed that shrimp Hsf1 facilitated WSSV infection in shrimp by suppressing host antiviral immunity. This mechanism highlights the significance of this transcription factor in viral infections. Nevertheless, the transcription factor activity of Hsf1 and h-HSF1 is always the basis for their involvement in facilitating viral infections in these studies, although the specific mechanism varies depending on the nature and context of the host-virus interaction.

We found that ammonia not only upregulated the expression of shrimp Hsf1 but also induced Hsf1 activation, which is essential for Hsf1 activity. In resting cells, h-HSF1 is usually bound to inhibitors and retained in its inactive form in the cytoplasm (11). Several mechanisms have been proposed for h-HSF1 activation, including the release of free h-HSF1, post-translational modifications, trimerization, and nuclear translocation (13, 41). Although we observed that the translocation of shrimp Hsf1 was enhanced by ammonia stress, we currently do not know how the translocation of Hsf1 into nucleus was induced in shrimp. Nevertheless, we showed that this induction relies on calcium-dependent p38 MAPK signaling. This mechanism is similar to those observed in other organisms. In mammals and fish, excess ammonium can displace potassium and activate N-methyl-d-aspartate-type glutamate receptors, leading to the influx of excess calcium (42, 43). Excess intracellular calcium activates multiple intracellular signal transduction pathways, including the calcium-dependent PKC/p38 MAPK pathway (44). Moreover, human p38γ can catalyze S326 phosphorylation, which is essential for the trimerization and nuclear translocation of h-HSF1 (45). Notably, this phosphorylation site is also present in shrimp Hsf1. In addition, we showed that chelation of intracellular calcium after ammonia stress inhibited p38 signaling, Hsf1 translocation into the nucleus, and the induction of Hsf1-regulated genes. Taken together, these findings suggest the presence of a conserved ammonia/calcium/p38 regulatory axis in shrimp.

In summary, we showed that ammonia stress suppresses shrimp antiviral immunity by targeting the interferon-like system and that Hsf1 is a central regulator of this process (Fig. 8E). Ammonia is the end product of protein catabolism and is derived from feces and unconsumed food; therefore, it constantly threatens shrimp health and growth, despite frequent water replenishment in aquaculture. This study provides a theoretical basis for water management to prevent stress-induced diseases and treat pathogen-associated diseases. As the activation of Hsf1 for stress relief is conserved among eukaryotes from yeast to humans and generally occurs under multiple different environmental stress conditions, this study may provide an explanation for why animals living in a stressed environment are more susceptible to pathogens than those living in unstressed conditions. Moreover, the current finding that the key stress relief factor Hsf1 affects host immunity also highlights the complexity of achieving homeostasis under stressful conditions.

## MATERIALS AND METHODS

### Animals and virus inoculum preparation

Healthy kuruma shrimp with a body weight of 3–5 g each were obtained from a breeding aquaculture farm in Jimo, Shandong, China. PCR was performed using DNA extracted from the hemolymph of randomly selected shrimp as a template to confirm that the shrimp were WSSV negative using the primers listed in Table S1. Thereafter, the shrimp were cultured in aerated seawater at 25°C and fed a commercial diet daily in the laboratory. Animals were randomly selected for the experiments. All animal experiments were approved by the Animal Ethical Committee of the Shandong University School of Life Sciences (permit number: SYDWLL-2021-98).

The viral strain used in this study was a gift from the East China Sea Fishery Institute (Shanghai, China) (31). To prepare successive inocula, red swamp crayfish were intramuscularly injected with the original inoculum. The gills of moribund crayfish were collected and homogenized manually using a glass homogenizer in phosphate-buffered saline (PBS; 140 mM NaCl, 2.7 mM KCl, 10 mM Na_2_HPO_4_, and 1.8 mM KH_2_PO_4_, pH 7.4) at a ratio of 1:10 (wt/vol). The homogenate was repeatedly frozen and thawed and centrifuged at 3,000 × *g* for 10 min at 4°C. The resultant supernatant was filtered through a 0.45-µm filter. After determining the viral titer of the filtrate, as described below, it was diluted to the appropriate titer with PBS and used as the viral inoculum.

### Ammonia treatment and immune challenge

After adapting to laboratory conditions for 1 week, the shrimp were transferred to new tanks containing seawater at certain concentrations for ammonia treatment. An ammonia gradient was generated by diluting an NH_4_Cl stock solution in fresh seawater with 1 mg/L of NH_4_Cl, equivalent to 0.262 mg/L of total ammonia nitrogen (TAN). The water was replaced daily to ensure that the TAN concentration remained constant. Immune challenge was performed 12 h after transfer by injecting the viral inoculum into the shrimp at a dose of 5 × 10^5^ viral copies. For the survival assays, 10^6^ viral copies per shrimp sample were used.

### Sample collection

Total RNA, protein, and genomic DNA samples were collected separately from tissues to evaluate gene transcription, protein levels, and viral loads. At least five shrimp were used to prepare each sample. Shrimp hemolymph was collected into an equal volume of pre-cooled anticoagulant (0.45 M NaCl, 10 mM KCl, 10 mM EDTA, and 10 mM HEPES, pH 7.45) and centrifuged at 800 × *g* for 7 min at 4°C to isolate the hemocyte pellet. Other tissues, including the heart, hepatopancreas, gills, stomach, and intestines, were also collected. Total RNA from each tissue sample was extracted using the TRIzol reagent (Invitrogen, Carlsbad, CA, USA; 15596026) and used as a template to synthesize first-strand cDNA using the ReverTra Ace qPCR RT Kit (Toyobo, Osaka, Japan; FSQ-101).

Proteins in the shrimp gills were extracted by homogenizing the tissue with PBS using an electric tissue homogenizer (Jingxin, Shanghai, China; JXFSTPRP-24L). The tissue homogenate was centrifuged at 12,000 × *g* for 10 min, and the supernatant was collected. After determining the protein concentration using a Bradford protein assay kit (Sangon, Shanghai, China; C503031), the supernatant was mixed with protein-loading buffer (Sangon; C508320), boiled for 5 min, and centrifuged at 8,000 × *g* for 3 min. The resulting samples were subjected to electrophoresis and western blot analyses.

Genomic DNA was extracted from the gill tissue homogenate using MagExtractor Genome (Toyobo; NPK-101), according to the manufacturer’s instructions. DNA concentration was determined using a NanoDrop 2000 spectrophotometer (Thermo Fisher Scientific, Waltham, MA, USA).

### Quantitative PCR

qPCR was performed to quantify the viral load or detect gene expression levels using iQ SYBR Green Supermix (170-8882, Bio-Rad, Hercules, CA, USA) and a CFX96 Real-Time System (Bio-Rad). The primers used are listed in Table S1. For viral quantification, a standard plasmid was constructed by ligating the WSSV vp28 fragment into the pBlueScript vector. After determining the concentration of the plasmid sample using a Nanodrop ND 2000 spectrophotometer (Thermo Fisher Scientific), it was serially diluted and detected by PCR. Genomic DNA was detected simultaneously in the shrimps. The PCR data for the plasmid samples were used to generate a standard curve, which was used to quantify the viral load in the test samples. For gene expression analysis, *β-actin* was detected simultaneously as the internal reference. The PCR data were processed using the 2^−ΔΔCT^ method (46). In all bar chart figures of gene expression, the expression levels are relative to those in the first column.

### Antibody preparation

Sequences encoding shrimp Hsf1, p38, and MjVago-L were amplified using primers listed in Table S1 and ligated into the pET32a (+) vector. The recombinant vectors were transformed into *Escherichia coli* Rosetta (DE3) for expression under induction by 0.2 mM isopropyl-b-D-thiogalactopyranoside at 28°C for 6 h. The recombinant proteins were purified using Ni-NTA His-binding resin (Merck, Darmstadt, Germany; 70666), eluted with 250 mM imidazole, and dialyzed against PBS to remove imidazole. The recombinant protein solution (1 mg/mL, 1 mL) was fully emulsified with an equal volume of complete Freund’s adjuvant (Sigma-Aldrich, St. Louis, MO, USA; F5881). The mixture was subcutaneously injected into New Zealand white rabbits. The second immunization was performed 25 days later, and the complete adjuvant was replaced with an incomplete adjuvant (Sigma-Aldrich; F5506). The rabbit antiserum was collected 7 days later. The antibodies were purified using a protein A resin column (GenScript, Nanjing, China; L00210) according to the manufacturer’s instructions. Antibody concentration was determined using a Bradford protein assay kit (Sangon; C503031), and the antibodies were stored at −80°C until use in the following studies. The specificities of these antibodies are presented in Fig. S7. The antibodies against Dorsal, Stat, VP28, and β-actin were generated as described in previous studies in which the specificities of these antibodies were shown (30, 31, 47, 48).

### Western blotting

Western blotting was performed to evaluate the protein levels. Protein samples, prepared as described above, were separated using sodium dodecyl-sulfate polyacrylamide gel electrophoresis (100 μg of total protein per lane) and then transferred onto nitrocellulose membranes using a Jim-X Semi-Dry Blotter (Jim-X, Dalian, China). The membranes were blocked using 5% non-fat milk dissolved in Tris-buffered saline (TBS: 150 mM NaCl and 10 mM Tris-HCl, pH 7.5) for 30 min and then incubated with specific antisera or antibodies overnight at 4°C. After washing three times with TBST (TBS with 0.05% Tween-20) and once with TBS, the membranes were incubated with secondary antibodies for 2–3 h at room temperature. The membranes were then washed three times with TBST and once with TBS. Immunoreactive signals were visualized using a high-signal enhanced chemiluminescence western blotting substrate (Tanon, Shanghai, China; 180-5001) and a 5200 Chemiluminescence Imaging System (Tanon).

The rabbit antisera against VP28, Hsf1, MjVago-L, β-actin, and p38 produced in our laboratory were used at a 1:500 dilution. Cross-reacting rabbit anti-human histone H3 polyclonal antibodies (ProteinTech, Wuhan, China; 17168-1-AP) were used to detect shrimp H3 expression at 1:1,000 dilution, and anti-phospho-p38 MAPK-T180/Y182 rabbit polyclonal antibodies (ABclonal, Woburn, MA, USA; AP0526) were used to analyze shrimp p38 phosphorylation at 1:1,000 dilution. Horseradish peroxidase-conjugated goat anti-rabbit secondary antibodies (Zhongshan, Beijing, China; ZB-2301) were used at a 1:10,000 dilution. All antisera and antibodies were diluted in 5% non-fat milk.

### RNA-seq analysis

RNA-seq analysis was performed to identify the genes whose expression was regulated by ammonia stress or ammonia-induced Hsf1.

To identify ammonia-regulated genes, shrimp were maintained in normal seawater or seawater containing 5 mg/L TAN for 12 h. Each group contained 30 shrimp. Three independent replicates were performed for each experiment. Total RNA was extracted from hemocytes and gills and sent for commercial sequencing by Biomarker Technologies (Beijing, China). A Bioanalyzer 2100 system (Agilent, Santa Clara, CA, USA) was used to determine the concentration and integrity of RNA samples. Only samples with RNA integrity values > 8 were used. Equal amounts of total RNA (0.375 μg) from the hemocytes and gills of each independent experiment were mixed. mRNAs were isolated, fragmented, and used as templates to synthesize first- and double-stranded cDNA. The cDNA was amplified by PCR and denatured to generate single-stranded cDNA. The cDNA library was sequenced using a NovaSeq 6000 platform (Illumina, San Diego, CA, USA). The raw reads were filtered by removing the reads containing adaptor sequences, with more than 10% of unknown nucleotides, or with more than 50% of low-quality bases (quality score  ≤ 10), to obtain high-quality clean reads using Cutadapt and in-house perl script (49). Clean reads were mapped to the kuruma shrimp genome (www.ncbi.nlm.nih.gov/assembly/GCA_017312705.1) using HISAT2 (50). Alignments were passed through StringTie (51) for transcript assembly. The assembly, together with the estimation of the abundance of each gene and isoform, was performed separately for each sample. After assembling each data set, the full set of assemblies was passed to the merging module of StringTie to generate a set of transcripts that were consistent across all samples for subsequent comparisons. The merged transcripts were fed back into StringTie to re-estimate the transcript abundance. The FPKM value was used to represent gene expression levels in each sample. The DESeq2_EBSeq tool was used to identify the DEGs. Valid expression of a gene was accepted when its FPKM value was greater than 1.5 in each sample. Differential expression was accepted at a cutoff fold change of ≥2 and adjusted false discovery rate (FDR) of ≥0.01. Gene annotations were also provided for the genome. New transcripts that were not previously annotated were analyzed and annotated using DIAMOND (52). BMKCloud (www.biocloud.net) was used to predict potential transcription factors and generate a heatmap.

To identify the genes whose expression was regulated by ammonia-induced Hsf1, ammonia stress treatment was performed 36 h after ds RNA injection. RNA was extracted 12 h later, and RNA-seq was performed at the Beijing Genomics Institute (BGI, Shenzhen, China) using the BGISEQ 500 system (BGI). The sequencing and data processing were performed similarly to the above procedure.

### RNA interference

DNA fragments of the target genes were produced by PCR amplification using specific primers linked to the T7 promoter (Table S1) and were then used as templates for dsRNA synthesis. An *in vitro* T7 Transcription Kit (Vazyme, Nanjing, China; TR102) was used to synthesize dsRNA according to the manufacturer’s instructions. dsRNA (50 μg) specific for a certain gene was injected into the shrimp hemocoel, with equal amounts of green fluorescent protein dsRNA as a control. The dsRNA injection did not significantly affect the total hemocyte count in shrimp (Fig. S8A). RNAi efficiency was determined 48 h after dsRNA injection by qPCR (Fig. S8B). After evaluating the RNAi efficiency, ammonia stress treatment was performed 36 h after dsRNA injection, and WSSV infection was performed 12 h later.

### Immunocytochemistry assay

Immunocytochemical analysis was performed to determine the subcellular distribution of transcription factors (31). At 24 h after WSSV infection, hemolymph was collected in cold anticoagulant containing 4% paraformaldehyde, fixed for 10 min, and centrifuged to obtain hemocytes. The hemocytes were washed three times with PBS, resuspended in PBS, and smeared onto glass slides pre-coated with poly-L-lysine. After attachment to the slides, hemocytes were washed three times with PBS and incubated with 0.2% Triton X-100 (in PBS) for 10 min. After three more washes with PBS, the hemocytes were blocked with 3% bovine serum albumin (BSA, in PBS) at 37°C for 1 h. Thereafter, anti-Dorsal or anti-Stat antibodies generated in our laboratory (1:1,000 diluted in 3% BSA) were added to the slides, and they were incubated overnight at 4°C (30, 31). After washing, the hemocytes were incubated with an Alexa-Fluor-488-conjugated goat anti-rabbit antibody (Abbkine, Wuhan, China; A23220; diluted 1:1,000 in 3% BSA) for 2 h. After washing, 4′,6-diamidino-2-phenylindole (AnaSpec, San Jose, CA, USA; AS-83210) was added to the slides for 10 min to stain the nuclei. After washing, the hemocytes were observed under an LSM 900 confocal microscope (Zeiss, Oberkochen, Germany). The captured images were analyzed using the ZFN software (Zeiss). To ensure comparability, all parameters for microscopic image capture were the same among the samples. The antibody, which was purified from the rabbit that did not receive the inoculum injection and was reared under the same conditions as the inoculum-injected rabbits, did not react with the shrimp protein samples and was used as a negative control (Fig. S3C and D).

### Chromatin immunoprecipitation assay

The ChIP assay was performed to detect the transcriptional regulation of *MjVago-L* and *MjFicolin* by Dorsal and Stat, respectively, using a ChIP assay kit (Beyotime, Jiangsu, China; P2078) according to the manufacturer’s instructions. Twenty-four hours after WSSV infection, gill extracts were pooled for the ChIP assay. PCR was performed to detect the amount of specific DNA fragments in the immunoprecipitates using the primers listed in Table S1.

### MjVago-L restoration experiment

To determine the ability of MjVago-L to restore ammonia-enhanced WSSV infection, recombinant (r) MjVago-L, produced as previously described (31), was injected into shrimp hemocoels along with the viral inoculum. An equal amount (5 μg) of the recombinant fusion tag was used as the control. Thereafter, *MjFicolin* transcription, VP28 levels, and shrimp survival rates were determined.

### Application of inhibitors

All inhibitors used in this study, including the p38 inhibitor SB203580 (S1076), JNK1/JNK2/JNK3 inhibitor SP600125 (S1460), and ERK1/2 inhibitor U0126 (S1102), were purchased from Selleck Chemicals (Houston, TX, USA) and dissolved in water containing 5% dimethyl sulfoxide (DMSO) and 30% polyethylene glycol 300 to a final concentration of 10 mM. These inhibitors were administered to shrimp by injecting 20 µL of inhibitor solution, with an equal volume of water/dimethyl sulfoxide solvent as the control. The injection was performed 30 min after the shrimp were transferred to the ammonia stress treatment group. WSSV infection was performed after 12 h.

### Separation of nuclear and cytoplasmic proteins

To determine the levels of activated Hsf1 in the nucleus, nuclear proteins were extracted using a Nuclear Protein Extraction Kit (Solarbio, Beijing, China; R0050) (31). The gills were collected 2 h after p38 inhibitor administration and homogenized in a cytoplasmic protein extraction reagent containing 1 mM phenylmethylsulfonyl fluoride and a phosphatase inhibitor cocktail (AbMole Bioscience, Houston, TX, USA; M7528). The tissue homogenate was shaken for 20 s using a vortex mixer and placed on ice for 5 min. After four rounds of the above treatment, the homogenate was centrifuged at 13,000 × *g* for 20 min at 4°C. The resultant pellet was washed three times with PBS and resuspended in a nucleoprotein extraction reagent containing 1 mM phenylmethylsulfonyl fluoride and a phosphatase inhibitor cocktail. The resuspension was processed by another four rounds of alternative shaking and ice bath incubation and then centrifuged at 13,000 × *g* for 20 min at 4°C. The obtained supernatant was used as the nuclear protein sample and was analyzed by western blotting using specific antibodies.

### Calcium detection

The shrimp were maintained in normal seawater or transferred to seawater containing 5 mg/L TAN. After transfer, the cell-permeating calcium chelator BAPTA-AM (Selleck; S7534) was injected into the shrimp hemocoel. The calcium content of shrimp gills was determined 30 min later. For the fluorescence probe assay, gills were collected and incubated with 3 µM of the calcium fluorescence probe, Fluo-3AM (Solarbio; F8840) for 30 min at 37°C. After washing with HEPES-buffered saline, gills were placed on slides containing glycerol. The slides were observed and imaged using an LSM 900 confocal microscope (Zeiss).

Calcium concentration was determined using a Calcium Colorimetric Assay Kit (Beyotime; S1063S), according to the manufacturer’s instructions. Briefly, gills were lysed using a lysate buffer. The lysate was centrifuged at 12,000 × *g* for 10 min at 4°C. The resulting supernatant was collected and incubated with the detection reagent at a ratio of 1:3. The incubation was continued for 10 min in the dark. The optical density at 575 nm was measured using a Multiskan FC microplate reader (Thermo Fisher Scientific).

### Quantification and statistical analysis

Co-localization analysis of the immunocytochemistry images was performed using the ImageJ software (NIH, Bethesda, MD, USA) at the Wright Cell Imaging Facility. Survival data were analyzed using the log-rank (Mantel–Cox) test using GraphPad Prism 8 software (GraphPad Inc., La Jolla, CA, USA). The expression profile data were analyzed using Student’s *t*-test. Differences were considered significant when the *P* value was less than 0.05.

## Data Availability

All relevant data are included in this study and the supporting material. Transcriptome data are available under BioProject accession numbers PRJNA1029798 and PRJNA1030072.

## SUPPLEMENTAL MATERIAL

Fig. S1 mbio.03136-23-s0001.tif Determination of appropriate concentration.

Fig. S2 mbio.03136-23-s0002.tif Expression profiles of shrimp Hsf1.

Fig. S3 mbio.03136-23-s0003.tif Immunofluorescence results.

Fig. S4 mbio.03136-23-s0004.tif Dorsal and stat blot data.

Fig. S5 mbio.03136-23-s0005.tif Significance of MjVago-L.

Fig. S6 mbio.03136-23-s0006.tif Activation of p38 by ammonia stress.

Fig. S7 mbio.03136-23-s0007.tif Specificities of the antibodies.

Fig. S8 mbio.03136-23-s0008.tif RNAi.

Supplemental legends mbio.03136-23-s0009.docx Legends for supplemental figures.

Table S1 mbio.03136-23-s0010.docx Primers.

ASM does not own the copyrights to Supplemental Material that may be linked to, or accessed through, an article. The authors have granted ASM a non-exclusive, world-wide license to publish the Supplemental Material files. Please contact the corresponding author directly for reuse.
