# Supplementary figures and images for: Ammonia stress-induced heat shock factor 1 enhances white spot syndrome virus infection by targeting the interferon-like system in shrimp
Source: mBio. 2024 Feb 15;15(3):e03136-23. doi: 10.1128/mbio.03136-23 (PMC10936208; doi:10.1128/mbio.03136-23)

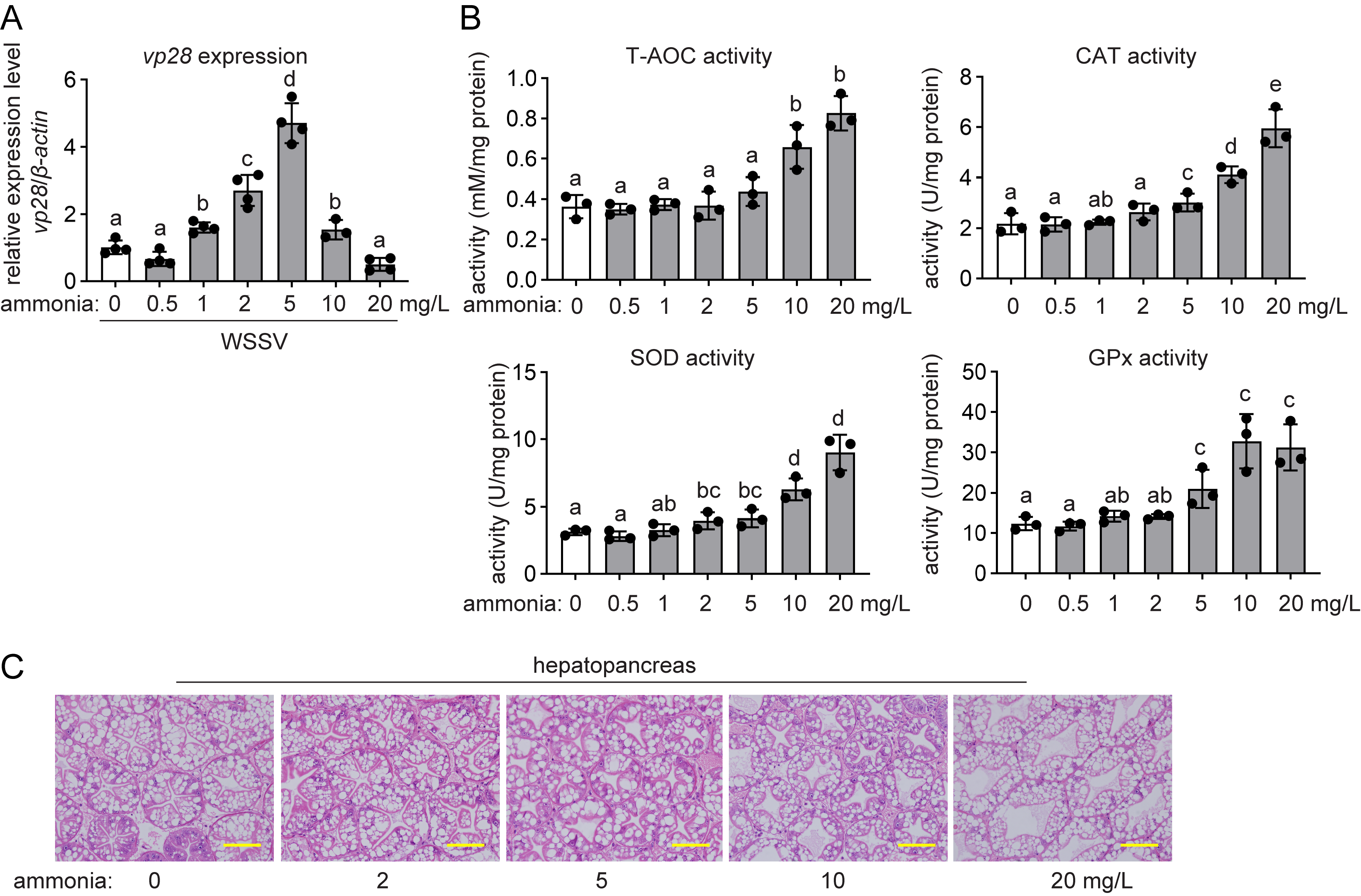

Supplement: Fig. S1 — Determination of appropriate concentration. [file mbio.03136-23-s0001.tif]

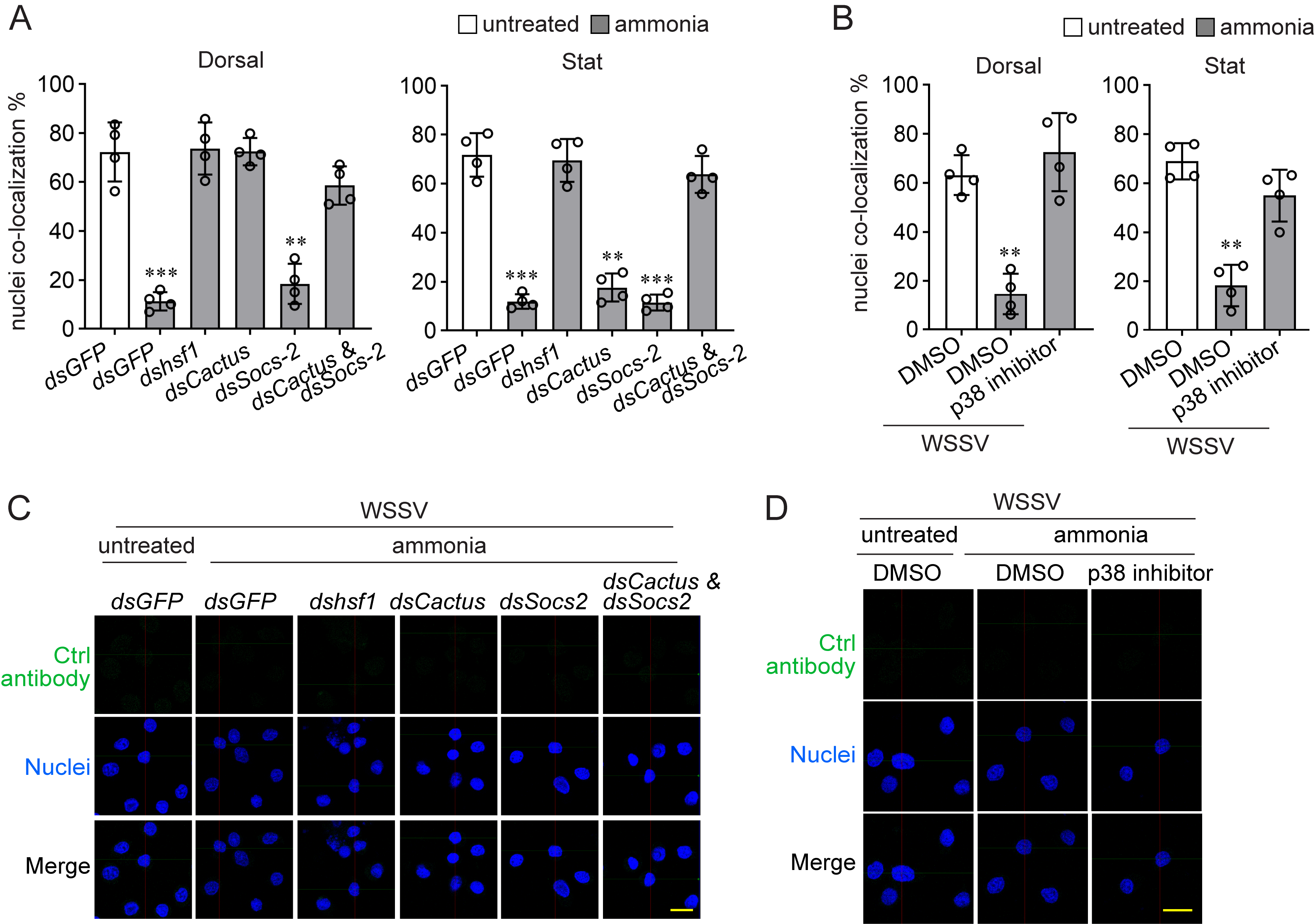

Supplement: Fig. S3 — Immunofluorescence results. [file mbio.03136-23-s0003.tif]

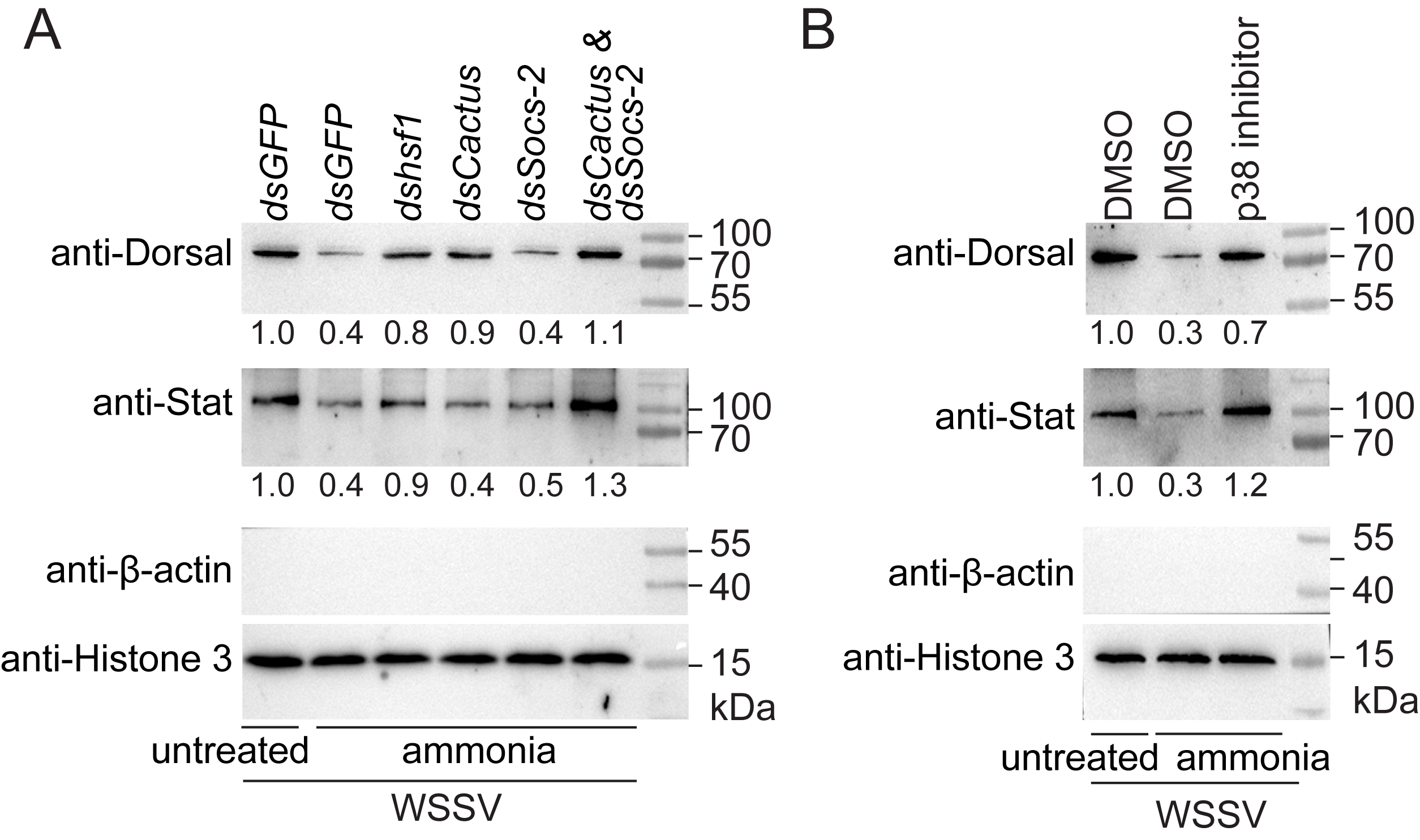

Supplement: Fig. S4 — Dorsal and stat blot data. [file mbio.03136-23-s0004.tif]

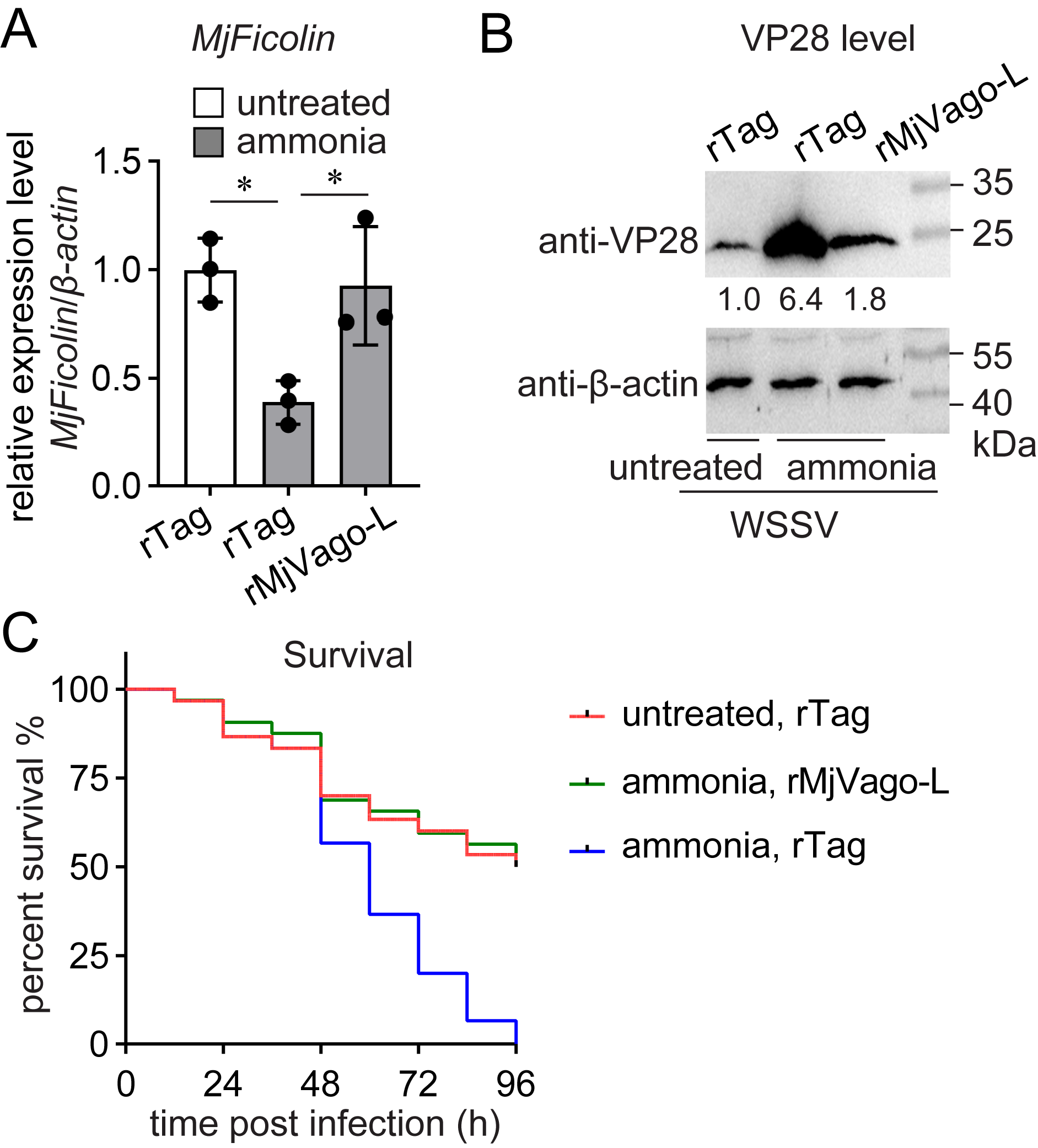

Supplement: Fig. S5 — Significance of MjVago-L. [file mbio.03136-23-s0005.tif]

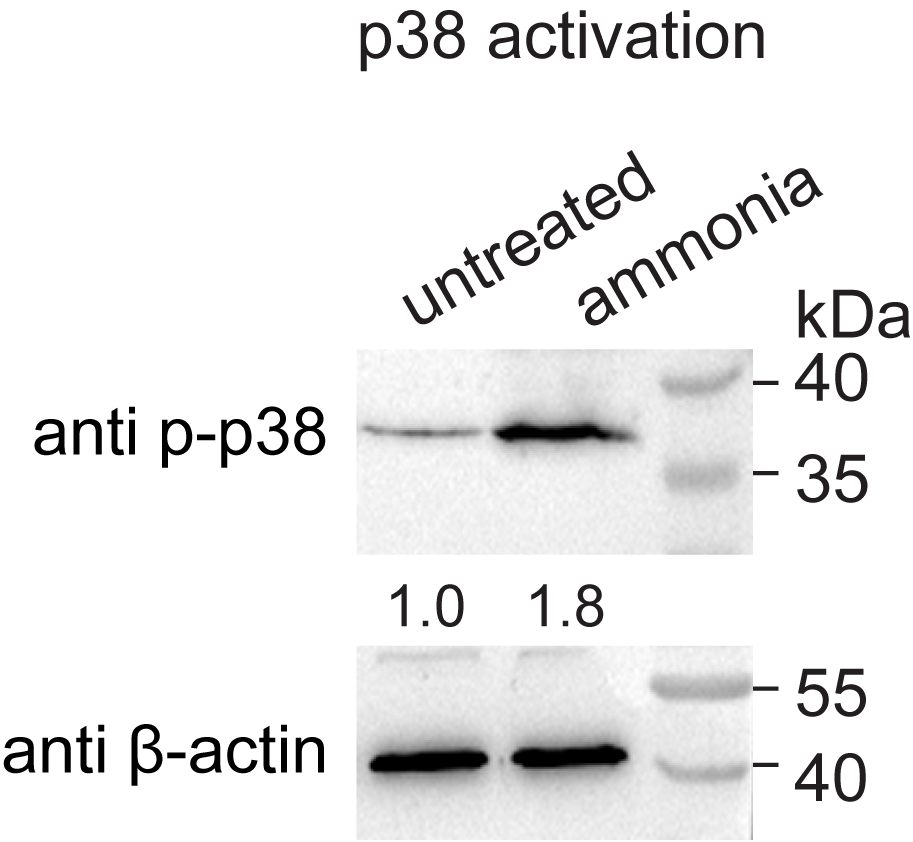

Supplement: Fig. S6 — Activation of p38 by ammonia stress. [file mbio.03136-23-s0006.tif]

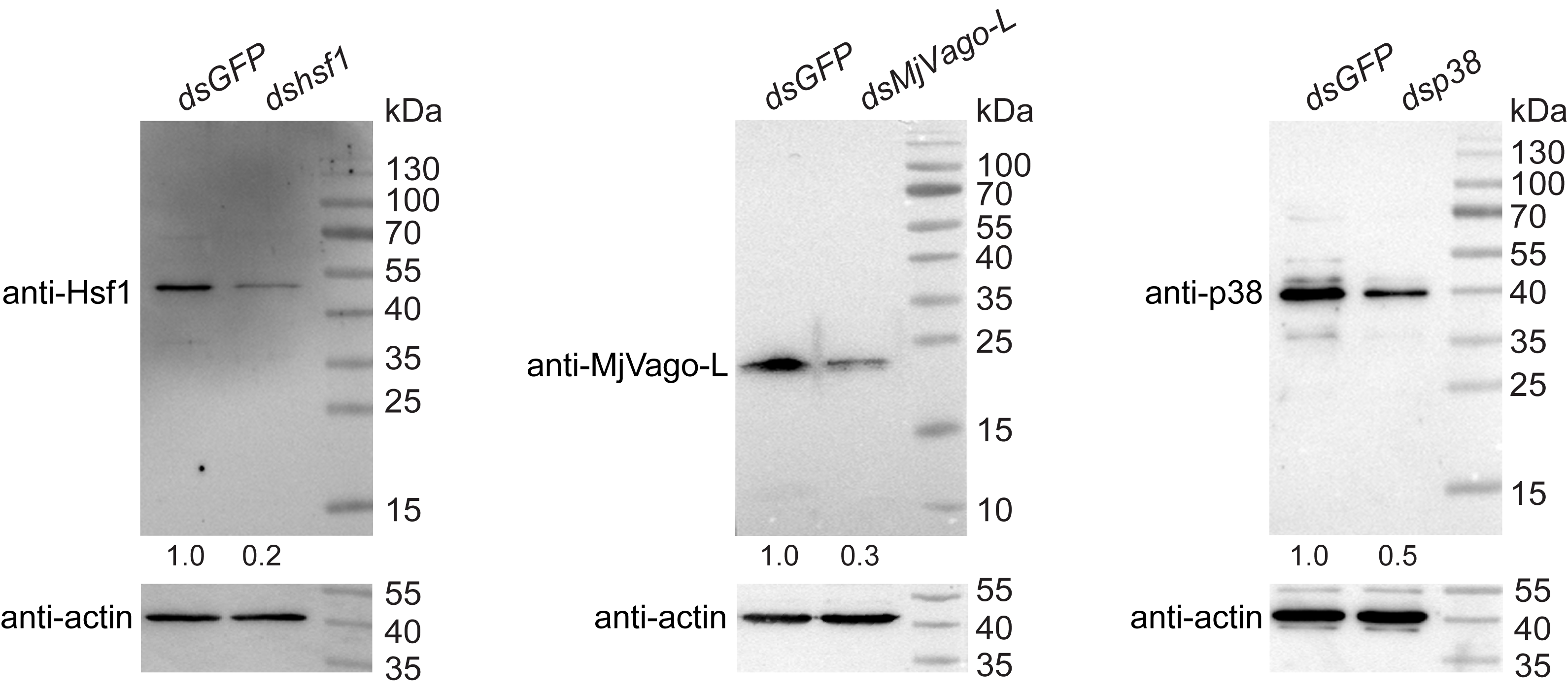

Supplement: Fig. S7 — Specificities of the antibodies. [file mbio.03136-23-s0007.tif]

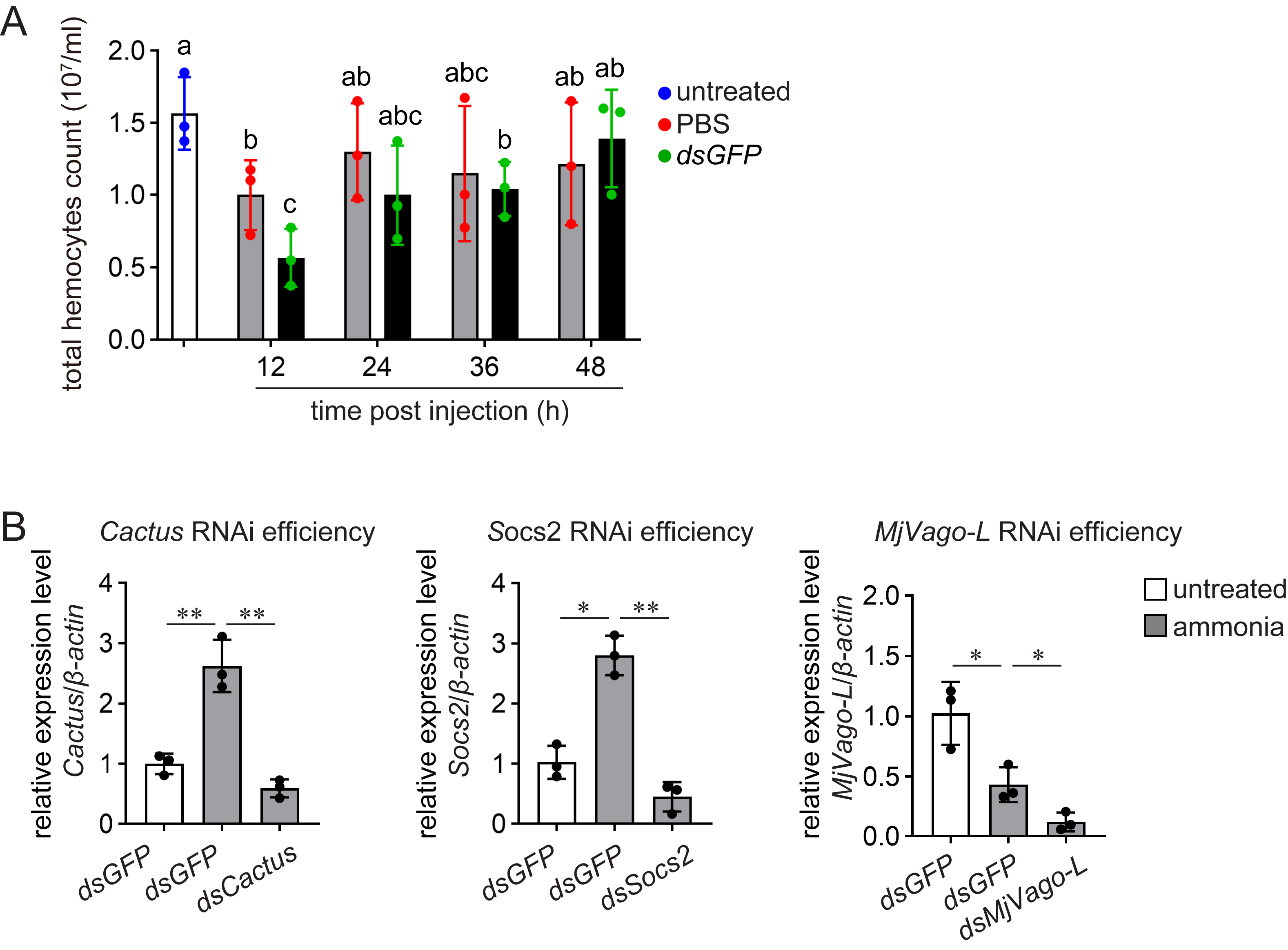

Supplement: Fig. S8 — RNAi. [file mbio.03136-23-s0008.tif]
