# Supplementary material for: Ammonia stress-induced heat shock factor 1 enhances white spot syndrome virus infection by targeting the interferon-like system in shrimp
Source: mBio. 2024 Feb 15;15(3):e03136-23. doi: 10.1128/mbio.03136-23 (PMC10936208; doi:10.1128/mbio.03136-23)
Supplement: Supplemental legends — Legends for supplemental figures. [file mbio.03136-23-s0009.docx]

**Fig. S1 Determination of 5 mg/L of ammonia as the appropriate concentration for stress treatment.**

(A) Influence of ammonia stress on *vp28* transcription. Shrimp were maintained in normal seawater or transferred into seawater containing different concentration of TAN. WSSV infection (5 × 10^5^ virions) was performed 12 h later. *vp28* expression was detected.

(B) Influence of by ammonia stress on hepatopancreas total antioxidant (T-AOC) capacity, catalase (CAT) activity, superoxide dismutase (SOD) activity, and glutathione peroxidase (GPx) activity. The hepatopancreas was collected after ammonia stress. Enzyme activities were determined using commercially available kits (Jiancheng, Nanjing, China; A015-2-1, A007-1-1, A001-3-2, and H545-1-1).

(C) Influence of ammonia stress on tissue morphology. Tissues were collected 24 h after ammonia stress, sectioned, and stained with hematoxylin and eosin. Scale bar = 20 μm.

All bar charts show the mean ± SD from three replicates. Different numbers indicate significant differences (*p* < 0.05) as determined by one-way analysis of variance (ANOVA). At least five shrimp were used to prepare each sample. Images are representative of three replicates.

**Fig. S2 Expression profiles of shrimp *Hsf1*.**

(A) Tissue distribution of *hsf1* mRNA. RT-PCR was performed with β-actin as an internal reference.

(B) Expression profiles of *hsf1* after ammonia stress treatment. Shrimp were cultured in normal seawater or seawater containing 5 mg/L TAN. *hsf1* expression levels in the indicated tissues were determined using quantitative qPCR.

(C) Expression profiles of *hsf1* after WSSV infection. Shrimp were cultured in normal water. WSSV infection (5 × 10^5^ virions) was administered, with PBS injection as control. *hsf1* expression was quantified in the gills using qRT-PCR.

The bar chart data are shown as the mean ± SD from three or four replicates. ∗∗∗ *p* < 0.001, ∗∗ 0.001 < *p* < 0.01, ∗ *p* < 0.05, as determined using Student’s *t* test. Electrophoresis images are representative of three independent replicates. At least five shrimp were used to prepare each sample.

**Fig. S3 Digitization and negative control of the immunofluorescence analysis.**

(A-B) Digitization of the immunofluorescence results.The colocalization percentage of Dorsal or Stat with nuclei was analyzed using ImageJ software. (A), relative to Fig. 6B; (B), relative to Fig. 7D. Data are the mean ± SD from four replicates.

(C) Negative control for Fig. 6B. Shrimp were injected with indicated dsRNAs, and stressed by ammonia 36 h later. WSSV infection was performed another 12 h later. The immunocytochemical assay was performed t using an antibody which does not react with any shrimp sample. Scale bar = 10 μm.

(D) Negative control for Fig. 7D. WSSV infection was performed at 0.5 h after inhibitor administration. The immunocytochemical analysis was performed at 24 h after WSSV infection using the control antibody. Scale bar = 10 μm.

**Fig. S4 Variation in the amount of nuclear Dorsal and Stat after the indicated treatments.**

(A) Regulation of the nuclear levels of Dorsal and Stat by ammonia-induced Hsf1-induced Cactus and Socs2. Shrimp were injected with the indicated dsRNAs, and stressed by ammonia 36 h later. WSSV infection was performed another 12 h later. The nuclear proteins were separated, and the amount of Dorsal and Stat in the nucleus was determined by western blotting using the indicated antibodies.

(B) Suppression of ammonia-reduced nuclear levels of Dorsal and Stat by p38 inhibition. Shrimp were maintained in normal seawater or subjected to ammonia stress treatment. The inhibitors were administered 30 min later. WSSV infection was performed another 12 h later. The amount of Dorsal and Stat in the nucleus was analyzed 24 h after WSSV infection.

Blot data are representative of three independent replicates. At least five shrimp were used to prepare each sample.

**Fig S5 Significance of MjVago-L in the sequential regulation downstream of ammonia-induced Hsf1.**

(A) Reversal of ammonia-suppressed *MjFicolin* expression by rMjVago-L administration. rMjVago-L (5 μg) was injected into shrimp hemocoels together with the viral inoculum. Gene expression was detected 24 h later. The data show the mean ± SD from three replicates. ∗ *p* < 0.05, as determined using Student’s t test.

(B) Suppression of ammonia-enhanced WSSV infection by rMjVago-L administration. rMjVago-L (5 μg) was injected into shrimp hemocoel together with the viral inoculum. VP28 levels were determined 24 h after WSSV infection. The blotting figures are representative of three independent replicates. At least five shrimp were used to prepare each sample.

(C) Inhibition of ammonia-induced shrimp death by rMjVago-L administration. rMjVago-L (5 μg) was injected into shrimp hemocoels together with the viral inoculum. The shrimp survival rate was recorded every 12 h. n = 30, log-rank (Mantel–Cox) test.

**Fig S6 Activation of p38 by ammonia stress.**

Shrimp were maintained in normal seawater or subjected to ammonia stress. Phosphorylated p38 was detected after 12 h. Figures represent three replicates. At least five shrimp were used to prepare each sample.

**Fig. S7 Specificities of the antibodies used in this study.**

Shrimp were injected with the indicated dsRNAs. The gills were collected and analyzed using the indicated antibodies. Blot data are representative of two replicates.

**Fig. S8 Side effect and knockdown efficiency of RNAi.**

(A) Influence of dsRNA injection on the total hemocyte counts of shrimp. Shrimp were injected with the indicated dsRNAs. Hemolymph was collected to determine the total hemocyte count using a hemocytometer. The data are shown as the mean ± SD from three replicates. Different characters above each column indicate significant differences, as analyzed by one-way ANOVA.

(B) RNAi efficiency of the indicated genes. Shrimp were maintained in normal seawater or transferred to seawater containing 5 mg/L TAN 36 h after dsRNA injection. Gene expression was detected after 12 h using qPCR.Data are shown as the mean ± SD from three or four replicates. ∗∗ 0.001 < *p* < 0.01, ∗ *p* < 0.05, as determined using Student’s *t* test. At least five shrimp were used to prepare the samples.

**Table S1 Primers used in this study.**
