# Supplementary material for: Ammonia stress-induced heat shock factor 1 enhances white spot syndrome virus infection by targeting the interferon-like system in shrimp
Source: mBio. 2024 Feb 15;15(3):e03136-23. doi: 10.1128/mbio.03136-23 (PMC10936208; doi:10.1128/mbio.03136-23)
Supplement: Table S1 — Primers. [file mbio.03136-23-s0010.docx]

**Table S1. Primers used in this study**

| Primers | Sequence (5’-3’) |
| --- | --- |
| **RT-PCR** |  |
| Hsf1 RTF | AGACCACCATTGACTCGC |
| Hsf1 RTR | AACCTCATTCCCACTTGC |
| Socs2 RTF | GAGTTCAGCGGCGTGGTA |
| Socs2 RTR | ACGGAGGCGTTGAGGGA |
| Cactus RTF | CCGCTGACCTAACCAACTATGA |
| Cactus RTR | TGCCGTCCGACCACTCTT |
| MjVago-L RTF | GGCGGAGGCAAAAGCATC |
| MjVago-L RTR | GTGGCGAGTGTCACCATAAGC |
| MjFicolin RTF | TACGAGGGCGATGCGAAA |
| MjFicolin RTR | CAACCACCACGATAGACGGA |
| β-actin RTF | CAGCCTTCCTTCCTGGGTATGG |
| β-actin RTR | GAGGGAGCGAGGGCAGTGATT |
| vp28 RTF | AGCTCCAACACCTCCTCCTTCA |
| vp28 RTR | TTACTCGGTCTCAGTGCCAGA |
| **RNAi** |  |
| Hsf1iF | GCGTAATACGACTCACTATAGGCCTT GAGCCTGAGACGAT |
| Hsf1iR | GCGTAATACGACTCACTATAGGGACTTGGACTTTGGTGGG |
| CactusiF | GCGTAATACGACTCACTATAGGGCGCAGGGCTGCTCGCCGCTGA |
| CactusiR | GCGTAATACGACTCACTATAGGGGAAGTAGCGATCTGCATTG |
| Socs2iF | GCGTAATACGACTCACTATAGGGATTCAAGATGGCCGATCCCGT |
| Socs2iR | GCGTAATACGACTCACTATAGGGTCCGTCTCAAGGGTTGTCTG |
| MjVago-LiF | GCGTAATACGACTCACTATAGGGGTCCTACTGTCAGGCACGAA |
| MjVago-LiR | GCGTAATACGACTCACTATAGGGCAAAAGTCATTGAATACCGAGA |
| GFPiF | GCGTAATACGACTCACTATAGGTGGTCCCAATTCTCGTGGAAC |
| GFPiR | GCGTAATACGACTCACTATAGGCTTGAAGTTGACCTTGATGCC |
| **ChIP** |  |
| MjVago-LChIPF | TACAAACACCGACAATGAGAAA |
| MjVago-LChIPR | GCGTACCGTAAACACTGAAGAT |
| MjFicolinChIPF | GATTAACAAAATGGACTTGCAG |
| MjFicolinChIPR | CCAACCATACTGACGCTCC |
